# Dissecting CD8+ T cell pathology of severe SARS-CoV-2 infection by single-cell immunoprofiling

**DOI:** 10.3389/fimmu.2022.1066176

**Published:** 2022-12-16

**Authors:** Felix Schreibing, Monica T. Hannani, Hyojin Kim, James S. Nagai, Fabio Ticconi, Eleanor Fewings, Tore Bleckwehl, Matthias Begemann, Natalia Torow, Christoph Kuppe, Ingo Kurth, Jennifer Kranz, Dario Frank, Teresa M. Anslinger, Patrick Ziegler, Thomas Kraus, Jürgen Enczmann, Vera Balz, Frank Windhofer, Paul Balfanz, Christian Kurts, Gernot Marx, Nikolaus Marx, Michael Dreher, Rebekka K. Schneider, Julio Saez-Rodriguez, Ivan Costa, Sikander Hayat, Rafael Kramann

**Affiliations:** ^1^ Institute of Experimental Medicine and Systems Biology, Medical Faculty, RWTH Aachen University, Aachen, Germany; ^2^ Department of Renal and Hypertensive Disorders, Rheumatological and Immunological Diseases (Medical Clinic II), Medical Faculty, RWTH Aachen University, Aachen, Germany; ^3^ Institute for Computational Biomedicine, Heidelberg University, Faculty of Medicine, Heidelberg University Hospital, Heidelberg, Germany; ^4^ Institute for Computational Genomics, Medical Faculty, RWTH Aachen University, Aachen, Germany; ^5^ Joint Research Center for Computational Biomedicine, RWTH Aachen University Hospital, Aachen, Germany; ^6^ Institute of Human Genetics, Medical Faculty, RWTH Aachen University, Aachen, Germany; ^7^ Institute of Medical Microbiology, Medical Faculty, RWTH Aachen University, Aachen, Germany; ^8^ Department of Urology and Pediatric Urology, RWTH Aachen University, Aachen, Germany; ^9^ Department of Urology and Kidney Transplantation, Martin Luther University (Saale), Halle, Germany; ^10^ Department of Medicine, St Antonius Hospital, Eschweiler, Germany; ^11^ Institute for Occupational, Social and Environmental Medicine, Medical Faculty, RWTH Aachen University, Aachen, Germany; ^12^ Institute for Transplantation Diagnostics and Cell Therapeutics, Medical Faculty, University Hospital Düsseldorf, Düsseldorf, Germany; ^13^ Department of Cardiology, Angiology and Intensive Care Medicine, Medical Faculty, RWTH Aachen University, Aachen, Germany; ^14^ Institute of Molecular Medicine and Experimental Immunology, Medical Faculty, University of Bonn, Bonn, Germany; ^15^ Department of Intensive and Intermediate Care, Medical Faculty, RWTH Aachen University, Aachen, Germany; ^16^ Department of Pneumology and Intensive Care Medicine, Medical Faculty, RWTH Aachen University, Aachen, Germany; ^17^ Institute of Cell and Tumor Biology, Medical Faculty, RWTH Aachen University, Aachen, Germany; ^18^ Department of Developmental Biology, Erasmus Medical Center, Rotterdam, Netherlands; ^19^ Department of Internal Medicine, Erasmus Medical Center (MC), Rotterdam, Netherlands

**Keywords:** SARS-CoV-2, scRNA-seq, scTCR-seq, immunoprofiling, CD8+ T cells, FCGR3A, CD16, NK-like T cell

## Abstract

**Introduction:**

SARS-CoV-2 infection results in varying disease severity, ranging from asymptomatic infection to severe illness. A detailed understanding of the immune response to SARS-CoV-2 is critical to unravel the causative factors underlying differences in disease severity and to develop optimal vaccines against new SARS-CoV-2 variants.

**Methods:**

We combined single-cell RNA and T cell receptor sequencing with CITE-seq antibodies to characterize the CD8^+^ T cell response to SARS-CoV-2 infection at high resolution and compared responses between mild and severe COVID-19.

**Results:**

We observed increased CD8^+^ T cell exhaustion in severe SARS-CoV-2 infection and identified a population of NK-like, terminally differentiated CD8^+^ effector T cells characterized by expression of *FCGR3A* (encoding CD16). Further characterization of NK-like CD8^+^ T cells revealed heterogeneity among CD16^+^ NK-like CD8^+^ T cells and profound differences in cytotoxicity, exhaustion, and NK-like differentiation between mild and severe disease conditions.

**Discussion:**

We propose a model in which differences in the surrounding inflammatory milieu lead to crucial differences in NK-like differentiation of CD8^+^ effector T cells, ultimately resulting in the appearance of NK-like CD8^+^ T cell populations of different functionality and pathogenicity. Our in-depth characterization of the CD8^+^ T cell-mediated response to SARS-CoV-2 infection provides a basis for further investigation of the importance of NK-like CD8^+^ T cells in COVID-19 severity.

## Introduction

More than two years into the pandemic, severe acute respiratory syndrome coronavirus type 2 (SARS-CoV-2) still poses significant challenges to the healthcare system, the economy, and the public. According to the WHO Coronavirus Dashboard there have been more than 590 million confirmed cases and more than 6.4 million deaths due to COVID-19 so far (August 2022) (1). Although some pharmacological therapeutic options are available today (2), the molecular mechanisms that drive the progression to a severe disease condition remain largely unknown, making vaccination an important instrument to prevent the occurrence of a life-threatening scenario. However, despite vaccine efficacy, particularly in preventing severe COVID-19 (3, 4), breakthrough infections occur repeatedly (5) and can result in severe disease (6–8) as well as serious long-term health consequences (9). Thus, a detailed understanding of the cellular and molecular mechanisms that determine the severity of SARS-CoV-2 infection is crucial to help developing targeted therapies and to improve vaccination strategies.

Much attention has been paid to the SARS-CoV-2-specific antibody response in the public debate, however, several studies point to a critical role of T cells (10), particularly CD8^+^ T cells, in controlling SARS-CoV-2 infection (11, 12). Impairments of the T cell compartment have been suggested to be involved in the pathogenesis of severe COVID-19. For instance, CD8^+^ T cell counts have been found to be substantially reduced in severe and fatal COVID-19 compared with mild cases (13). Additionally, hyperactivated CD8^+^ T cell states have been associated with COVID-19 lethality and severity (14–16) and CD8^+^ T cell exhaustion has been identified as a characteristic of severe SARS-CoV-2 infection (16).

Single-cell RNA-sequencing (scRNA-seq) techniques offer the opportunity to investigate cellular heterogeneity, to uncover cell type-specific gene expression and to dissect cell type-specific differences between disease conditions. Various studies have used scRNA-seq to investigate the innate and adaptive immune response in COVID-19 (17–24). Additionally, within recent years, scRNA-seq has led to the discovery of large phenotypic diversity within the CD8^+^ T cell compartment (25, 26).

In this study, we perform scRNA-seq combined with single-cell T cell receptor sequencing (scTCR-seq) and surface protein profiling using cellular indexing of transcriptomes and epitopes by sequencing (CITE-seq) (27) of CD8^+^ T cells in mild and severe COVID-19 and healthy controls. We observe large functional and phenotypic heterogeneity within the CD8^+^ T cell compartment and identify increased CD8^+^ T cell exhaustion in individuals with severe SARS-CoV-2 infection. By performing trajectory inference, we identify a terminally differentiated CD16^+^ CD8^+^ effector cell population with an NK-like phenotype that might be relevant in viral control. Deeper profiling of these NK-like T cells reveals heterogeneity within this population and substantial phenotypic differences between mild and severe disease. Thus, our study provides insights into a poorly described CD8^+^ T cell population that might be important for the antiviral response against SARS-CoV-2. Moreover, aberrant differentiation of this subpopulation might be crucially involved in the pathogenesis of severe SARS-CoV-2 infections.

## Materials and methods

### Study design

With this study we wanted to dissect the differences in CD8^+^ T cell responses between individuals with mild and severe SARS-CoV-2 infection at a single-cell resolution. We performed scRNA and TCR-seq to identify differences in gene expression characteristics and clonal expansion between CD8^+^ T cells from the different disease conditions. Additionally, we used CITE-seq antibodies to identify functional subgroups of CD8^+^ T cells and to perform a surface-protein profiling.

### Patient recruitment and clinical data

Laboratory-confirmed COVID-19 patients were recruited from the University Hospital of the RWTH Aachen University and from the Sankt Antonius Hospital Eschweiler from May to September 2020. All patients provided informed consent and the study was performed in accordance with the Declaration of Helsinki. For patients who were not able to give consent themselves, their legal representative agreed to their participation in the study. The study protocol was reviewed and approved by the Ethical Board of the RWTH Aachen University Hospital (vote: EK 078/20). Initially, 12 COVID-19 patients (6 with mild COVID-19 and 6 with severe COVID-19) and 3 healthy volunteers were included in the study. After initial processing of the single-cell data, we observed an extremely high proportion of effector cells in one healthy control sample. In addition, the respective healthy volunteer reported an unclear infection in early January 2020, approximately four months before the start of the study. Since we could not rule out that the patient had been infected with SARS-CoV-2, we excluded this sample in order to avoid a bias. Thus, the final analyses were carried out on samples from 6 individuals with mild COVID-19, 6 samples with severe COVID-19 and 2 control samples. Patients were pseudonymized and clinical as well as epidemiological data were obtained from the electronic hospital information system “CGM Medico”. Clinical data is provided in **Table S2**.

### Group allocation

Based on the clinical course, patients were divided into two major groups: severe or mild SARS-CoV-2 infection. Patients with asymptomatic infection and symptomatic patients who did not require mechanical ventilation were allocated to the group of mild infection. Symptomatic patients who required mechanical ventilation were allocated to the group of severe SARS-CoV-2 infection. Group allocation for each patient is shown in **Table S2**.

### Sample collection and PBMC isolation

10-30 ml of blood per patient were collected in either 9 ml S-Monovettes, K3 EDTA 92x16 mm, or in 5.5 ml S-Monovettes, 75x15 mm, provided by Sarstedt (Nümbrecht, Germany). The samples were stored at 4°C for a maximum of 7 hours until further processing. Peripheral blood mononuclear cells (PBMCs) were isolated by Ficoll gradient centrifugation. The isolated PBMCs were resuspended in 10% DMSO in FCS and immediately frozen gradually. The frozen PBMC samples were stored at -152°C. In addition, serum samples were frozen for each patient. After thawing, the PBMCs were immediately diluted with 10 ml 5% FCS in PBS and centrifuged at 500 rcf for 5 minutes. The supernatant was removed and the PBMCs were resuspended in 5 ml 5% FCS in PBS and filtered through a 20 µm pluriStrainer**^®^** provided by pluriSelect (Leipzig, Germany) to obtain a single-cell solution.

### SARS-CoV-2 antibody testing

To exclude healthy controls with a previous SARS-CoV-2 infection, we tested the subjects for SARS-CoV-2-specific IgG antibodies by performing the Euroimmun anti-SARS-CoV-2 ELISA (IgG) (EUROIMMUN, Lübeck, Germany) (**Table S2**). An IgG ratio of > 2.5 was considered a positive test, a ratio between 0.8 and 2.5 was considered an intermediate result and a ratio < 0.8 was considered a negative test (28).

### Single-cell immune profiling of CD8^+^ T cells

PBMC samples were thawed as described above. After the PBMCs were passed through a strainer, a second centrifugation was performed. The supernatant was removed and cells were diluted in 100 µl 5% FCS in PBS. 5 µl Human TruStain FcX (BioLegend) was added and incubated for 10 minutes at 4°C. The panel of 15 TotalSeq-C antibodies (BioLegend) was pooled, using 0.5 µg of each antibody (**Table S1**). Samples were incubated with MHC class I Dextramer reagents provided by Immudex (Copenhagen, Denmark) for 10 minutes at 4°C, followed by a 30 minute incubation with the TotalSeq-C antibody pool and 5 µl of a PE/Cyanine7 anti-human CD8 antibody (BioLegend) at 4°C. Cells were washed two times using 3 ml 5% FCS in PBS and rediluted in 2 ml 5% FCS in PBS. For the detection of dead cells, DAPI was added at a final concentration of 0.5 µg/ml. Cells were sorted into 1% BSA in PBS on a Sony Cell Sorter by gating on two populations; a PE^+^ population (MHC I Dextramer reagent-positive) to enrich for SARS-CoV-2-specific T cells and a CD8^+^ PE^-^ fraction (**Figure S1A**). If the number of PE^+^ cells did not exceed 10,000 cells, a maximum of 3,000 CD8^+^ PE^-^ cells were added prior to single-cell partitioning and barcoding *via* Chromium Controller (10x Genomics). For each sample, three libraries were prepared; a 5´ gene expression library (GEX), a T cell receptor enriched library (VDJ), and a surface protein library containing the TotalSeq-C barcodes (ADT). After fluorometric quantification, the libraries were pooled in a 5:1:1 ratio for the GEX library, the VDJ enriched library and the ADT library, respectively. The pooled libraries were again quantified using a Quantus™ Fluorometer (Promega, Madison, Wisconsin, USA) and sequenced on a NextSeq 500 platform (Illumina, San Diego, CA, USA) with 2x 150 cycles.

### Single-cell RNA seq data processing

Raw scRNA-seq FASTQ files were aligned to the human GRCh38 genome with *Cell Ranger* (4.0.0) with default settings (10x Genomics). For every patient, the paired GEX and ADT libraries were processed together with the *count* function and the VDJ enriched library was processed separately with the *vdj* method. Downstream analysis was conducted with *Seurat* (4.0) (29) in *R* version 4.0.3. Cells with < 200 or > 3,000 detected genes and more than 10% mitochondrial read content were filtered out (**Figures S1H–J** are referred to for scRNA quality control metrics per sample). The GEX and ADT assays were log and centered log ratio (CLR) normalized, respectively, and were subsequently scaled with default settings.

### Clustering and cell annotation

In order to cluster and characterize cell subtypes, the samples were integrated based on the GEX libraries for a first round of clustering. For each sample, the top 2,000 most variable genes were obtained using the *FindVariableFeatures* function in Seurat *version 4.0* and dimensional reduction was performed on the variable features with a principal component analysis (PCA). To account for batch-effects, the samples were integrated using the *harmony* algorithm version 0.1.0 (30) with default setting, ‘sample’ as the batch variable, and the data was embedded in a Uniform Manifold Approximation and Projection (UMAP) using 30 principal components. A nearest neighbor graph was built with 30 principal components using *FindNeighbors* and unsupervised clustering was performed using a Louvain algorithm with *FindClusters* and a resolution of 1. In order to determine cluster-specific markers, a Wilcoxon rank sum test was performed with *FindMarkers* using min.pct = 0.25. Only genes with a false discovery rate (FDR) < 5% were considered. High-level cell annotation of the clusters was performed on the integrated data followed by filtering of non-T cells and clusters consisting mainly of low-quality cells. A second round of clustering was performed as described above using a resolution of 0.5. Low-level annotation of the resulting clusters was based on a combination of GEX and ADT marker expression. One healthy sample was removed from the study as the volunteer informed us of an unknown infection in early January 2020 and exhibited high levels of differentiated effector T cells. Cell-cycle analysis was performed on the clusters using *CellCycleScoring* (**Figure S1G**) and mitochondrial gene content was computed per disease condition (**Figure S1J**). After the final round of annotation and after determining the average cell proportions per condition, we removed all cells that were annotated as MAIT cells, γδ T cells and atypical NKT cells to achieve a dataset of only CD8^+^ T cells. Cells that expressed either TRAV1-2, TRAJ33 or both, which are T cell receptor genes characteristic of MAIT cells, were also excluded from the analysis. Lastly, T cells with missing TCR-seq information (**Figure S1F**) were removed, resulting in a final dataset of 25,506 cells for all downstream analysis.

### Differential gene expression and gene set enrichment analysis

The final dataset consists of control (*n* = 2,086 cells), mild (*n* = 12,251 cells), and severe disease conditions (*n* = 11,169 cells). For functional characterization of the differences between the disease conditions, differential gene expression analysis was performed with *FindMarkers* using min.pct = 0.25 and FDR < 5%. When contrasting conditions, only cell types with counts > 20 in both groups were considered for the analysis. A pre-ranked gene set enrichment analysis was performed with the *fgsea* package (31). The gene sets C2, C5 (subcategory BP), C7, C8 and H were used for the analysis and were downloaded with the *msigdbr* package. Selected gene sets with an FDR < 5% were visualized as bar plots. A full list of significantly differentially expressed genes in each CD8^+^ T cell population and corresponding gene sets is provided in **Table S4**. Differentially expressed genes between disease conditions in CD16^+^ CD8^+^ T_EMRA_ subpopulations and associated gene sets for each test are listed in **Tables S6, S7**, respectively.

### Subclustering of CD8^+^ NK-like T_EMRA_ cells

For subclustering, CD8^+^ NK-like T_EMRA_ cells were subsetted using the *subset* function in *Seurat*. The subset was normalized, variable features were identified and the data was scaled following the standard *Seurat* workflow. Dimensional reduction was performed and the subset was re-integrated with the *harmony* algorithm version 0.1.0 using ‘patient’ as the batch variable. A shared nearest neighbor graph was built with 20 principal components using *FindNeighbors* and unsupervised clustering was performed on the re-integrated data using a Louvain-based algorithm with *FindClusters* and a resolution of 1. Marker genes were identified with *FindMarkers* using min.pct = 0.5 and FDR < 5%.

### Calculation of functional scores

Gene sets for the calculation of exhaustion and cytotoxicity scores were adopted from Baryawno et al. (32). For the estimation of an NK-like phenotype (NK cell signature score), we used a gene set derived from the cell type signature gene set ‘HAY_BONE_MARROW_NK_CELLS’ (33). For the calculation of an apoptosis score we used the ‘REACTOME_APOPTOSIS’ gene set, obtained from MSigDB. A score value for each gene set was calculated for each cell using the *AddModuleScore* function in *Seurat* and score values were plotted as violin plots using the *VlnPlot* function in *Seurat*. For the comparison of functional scores within CD16^+^ CD8^+^ T_EMRA_-1 cells between the mild and the severe disease condition, CD8^+^ NK-like T_EMRA_-1 cells were subsetted using the *subset* function in *Seurat*. Violin plots were created using the *geom_violin* function combined with the *geom_boxplot* function in the *ggplot2* package (3.3.6) and significance tests were performed using Wilcoxon test within the *stat_compare_means* function in *ggpubr* (0.4.0).

### Integration with reference CD8^+^ T cell dataset and reference mapping of exhausted and NK-like populations

To computationally validate our findings in a larger dataset, we subsetted the large reference dataset from Ren et al. (34) for all CD8^+^ T cells. We then generated two reference datasets. The first one was generated by filtering the CD8^+^ T cells for data that was generated from frozen PBMCs using 5´-sequencing to match the conditions of our dataset. This PBMC-derived reference CD8^+^ T cell dataset was used for investigation of exhaustion as well as for the integration with our dataset to validate the existence of an NK-like CD8^+^ T cell population. For integration, the CD8^+^ reference dataset was re-integrated with the *harmony* (0.1.0) algorithm using ‘patient’ as the batch variable and subsequently clustered using the Louvain algorithm implemented in *Seurat* (**Figures S3A–C**). Next, anchors were identified and the reference CD8^+^ T cell dataset was integrated with our dataset using *Seurat* (**Figures S3D–G**). The proportion of cells from our dataset (query) in the integrated clusters was calculated by dividing the absolute number of cells of a certain CD8^+^ T cell subtype in each integrated subcluster by the total count of cells of this subtype in our dataset. Results were visualized as heatmap using *pheatmap* package (1.0.12).

To isolate and subcluster NK-like CD8^+^ T cells in the reference dataset, cell IDs of all cells that clustered together with our CD8^+^ NK-like T_EMRA_ cells in the integrated dataset were isolated using the *WhichCells* function in *Seurat*. These cells were annotated as ‘NK_like_subset’ in the reference dataset based on cell ID. Further, all NK-like CD8^+^ T cells in the reference dataset were subclustered as described above (**Figures S7D–F**). The same cell ID-based approach was used to identify cells in the reference dataset that mapped to exhausted CD8^+^ T cells in our dataset.

Besides the PBMC-derived reference dataset, we also filtered the large CD8^+^ T cell dataset for samples derived from bronchoalveolar lavage fluid (BAL). The BAL-derived CD8^+^ T cell reference dataset was re-integrated as described above (**Figures S3H–J**) and used to investigate exhaustion in lung-derived CD8^+^ T cells.

### Mild and severe disease scores

To identify gene sets in NK-like CD8^+^ T cells that are related to disease severity, we performed differential gene expression analysis between all CD8^+^ NK-like T_EMRA_ cells from the mild and the severe condition using the *FindMarkers* function with a min.pct = 0.5 and FDR < 5%. Next, we identified genes differentially expressed between the two disease conditions, which overlapped with highly significant marker genes of our CD8^+^ NK-like T_EMRA_ population. To optimize identification of gene sets, the p-value threshold for the selection of CD8^+^ NK-like T_EMRA_ marker genes was adjusted multiple times. Finally, choosing only the most significant marker genes with a p-value threshold of 1e-17 yielded best results. Genes with an average log_2_-fold change > 0 in differential expression analysis between mild and severe COVID-19 were combined into the ‘mild disease score’, while genes with an average log_2_-fold change < 0 were combined into the ‘severe disease score’.

Mild and severe disease score values were calculated for every cell in our CD8^+^ NK-like T_EMRA_ cell subset as well as for every cell in the reference NK-like CD8^+^ T cell subset using *AddModuleScore*. Results were grouped by condition (query and reference) and by outcome (reference) and plotted as boxplots using *geom_boxplot* in *ggplot2* package (3.3.6). Lastly score values were calculated for every cell in our whole dataset as well as for every cell in the reference CD8^+^ T cell dataset and score values were projected onto the UMAP using the *FeaturePlot* function in *Seurat*. To investigate cell type-specific score values, score values were plotted per cell type using the *VlnPlot* function in *Seurat* (**Figures S7H, I**).

### Signaling pathway and transcription factor activity

Signaling pathway activities were estimated with PROGENy (35, 36) using the top 500 footprint genes per pathway. To test for significant differences between the conditions, Wilcoxon rank sum tests were performed on relevant pathways and cell types with FDR < 5%. Transcription factor activities were computed with the *viper* package (1.24.0) (37) using regulons with confidence levels A, B or C from DoRothEA (38). The changes in transcription factor activity were estimated per cell type using the condition contrasts obtained from differential gene expression analysis with *FindMarkers*. Transcription factor activities with FDR < 5% in pairwise comparisons between the conditions were visualized with *geom_tile* function in *ggplot2*. **Figure S6G** is referred to for all PROGENy pathway activities per condition.

### Data integration for cell-cell communication analysis

To estimate cell-cell interactions between our CD8^+^ T cell populations and non-T cell populations, our dataset was integrated with a selected subset of data from Ren et al. (34). For sample selection from the public dataset, we included 16 disease samples (mild/moderate = 4, severe/critical = 12) where matched samples from both PMBCs and bronchoalveolar lavage fluid (BAL) were available. Additionally, we included 6 healthy controls. In total, the subsetted public dataset consisted of 119,587 disease and 41,919 healthy cells (total *n* = 161,506) (**Table S10**).

Integration of our single-cell dataset with the public dataset was performed using *harmony* (0.1.0). Briefly, different samples were used as the batch variable to account for batch-effects between the two datasets. Network neighborhood algorithm, followed by the Louvian clustering approach implemented in *Seurat* (4.1.1), was used to identify cell-clusters in the integrated dataset. The final integrated dataset consisted of a total of 187,012 cells from 36 samples.

### Cell-cell communication

To predict cell-cell interactions, *LIANA* (0.1.6) (39) was used per disease condition with the *statistical_analysis* method. Cell types with counts > 20 per condition were included in the analysis and the log-normalized and scaled counts were used as input. Ligand-receptor interactions with p-value < 0.05 were considered for the downstream analyses.

*CrossTalkeR* (40) was used to compute changes in ligand-receptor interactions between the conditions. Briefly, *CrossTalkeR* constructs representations of the ligand-receptor networks for each condition, where the edges of the network are weighted by the number of interactions and the sum of weights of the interaction-pairs obtained by *LIANA*. Differential cell-cell interaction networks were constructed by subtracting the condition state network from the control states.

For the characterization of differences in interactions with CD8^+^ NK-like T_EMRA_ cells between the conditions, differential cell-cell interactions that were predicted by *CrossTalkeR* were filtered for CD8^+^ NK-like T_EMRA_ cells as *receptor* cluster, while all non-CD8^+^ T cells were considered as *ligand* cluster (except for megakaryocytes). Differential absolute LR-Scores were plotted as dot plots for selected interactions that have been shown to be relevant in NK cell development and function.

To focus on differences in selected interactions between mild and severe SARS-CoV-2 infection, all CD8^+^ T cell populations were regarded as *receptor* cluster, while the same cell types as before were regarded as *ligand* clusters. Boxplots displaying the LR-Scores for each predicted interaction, grouped by condition, were plotted using the *geom_boxplot* function in *ggplot2*. Wilcoxon-test was used to compare for statistical differences between the mild and the severe condition using the *stat_compare_means* function in *ggpubr*.

### Trajectory inference and pseudotemporal differential gene expression

In order to estimate CD8^+^ T cell differentiation, trajectory inference was performed with *Slingshot* (2.2.1) (41). In addition to the previously removed MAIT, ɣδ, NKT cell populations, CD8^+^ CD73^+^ T_reg_ populations were also excluded from the pseudotime analysis in order to only include T cell subpopulations likely to originate from the CD8^+^ naïve T cells. The final dataset for trajectory analyses consisted of 24,716 cells. *Slingshot* was run on the UMAP embedding of the remaining clusters and the CD8^+^ naïve T cell population was manually designated as the root of all inferred trajectories. Two trajectories were determined by the pseudotime analysis; the short-lived effector cell (SLEC) lineage and exhaustion (EX) lineage. In order to test for significant differences between the distribution of the mild and the severe disease conditions across pseudotime, a Kolmogorov-Smirnov test was performed for each lineage.

For temporal differential gene expression analysis between the two trajectories, *tradeSeq* (1.8.0) was used (42). A negative binomial generalized additive model (NB-GAM) was built on the 10,000 most variable genes and pseudotimes for the mild and severe conditions using the *fitGAM* function. In order to study differences in temporal gene expression between the conditions, a condition-specific smoother was computed per lineage. 6 knots were used for the NB-GAM (**Figure S4A** is referred to for a visualization of the knots projected onto the integrated UMAP). Differential gene expression between the progenitor and differentiated cell populations was performed with *startVsEndTest* using l2fc = log_2_(2). The significant genes from the test were modeled with *predictSmooth* using nPoints = 50 and visualized with *pheatmap*. The expression of significant genes across pseudotime was visualized with *plotSmoothers*. To characterize potential early drivers of differentiation towards the two trajectories, *earlyDETest* was used at the bifurcation point (between knots 2 and 3) with l2fc = log_2_(1.5). Differential gene expression between the end stages of the lineages was performed with *diffEndTest* using l2fc = log_2_(2). Temporal differential expression between the mild and severe conditions for each lineage was computed with *conditionTest* using l2fc = log_2_(2), global = TRUE, and pairwise = TRUE. For all tests performed with *tradeSeq*, only genes with FDR < 5% were considered. For each test performed with *tradeSeq*, all genes were ranked based on the estimated Wald statistic and a gene set enrichment analysis was performed as previously described. **Table S5** is referred to for a full list of significantly differentially expressed genes for each test.

### T cell receptor clonality analysis

In order to study T cell receptor (TCR) clonality in the scRNA data, TCR clonotypes were assigned based on the VDJ library using the *cellranger vdj* function. For the analysis, only MHC class I restricted T cell subtypes were considered. For clonality analysis in the CD8^+^ reference dataset, the data frame containing TCR-seq data was loaded into *R*. The reference dataset was filtered for cells for which TCR-seq data were available in the data frame and vice versa. Finally, TCR-seq data was added to the dataset using the *AddMetaData* function implemented in *Seurat*. The clonotypes were grouped based on the level of expansion and designated as; single (*n* = 1), small (1 < *n* ≤ 5), medium (5 < *n* ≤ 20), large (20 < *n* ≤ 100), or hyperexpanded (*n* > 100). The relative abundance of the clonotype size groups was computed for the conditions and cell types and visualized as bar charts. **Figures S5A, D, E** are referred to for the TCR clonotype size distribution for each cell type per condition in our dataset, the PBMC-derived, and the BAL-derived CD8^+^ reference datasets, respectively.

In order to estimate changes in TCR diversity between the disease conditions during CD8^+^ T cell differentiation, the Shannon diversity index was calculated over pseudotime for the SLEC and EX trajectories. Cells in the two trajectories were grouped into 8 different sets by ascending order of pseudotime. For each bin, the TCR chains were extracted along with the condition (mild or severe) and chain (TRA or TRB) level information. TCR sequences were rearranged into the ‘TCR_V_CDR3_TCR_J’ format for TRA and TRB chains, and subsequently these sequences were combined into a format ‘TRA_V_CDR3A_TRA_J_TRB_V_CDR3B_TRB_J’ to calculate the Shannon index based on the total TCR sequence information. Shannon entropy was then applied on the occurrence of several TCR chains by using the *vegan* package (2.6.2) (43).

To investigate overlap in TCR repertoires between the three conditions, TCR sequences were first rearranged into the ‘TCR_V_CDR3_TCR_J’ format. Overlap between the conditions was calculated and visualized separately for TCR alpha and beta chain with the *ggvenn* function in the *ggvenn* package (0.1.9).

To quantify clonotype similarity between CD16^+^ CD8^+^ T_EMRA_ subpopulations (**Figure S6C**), the Morisita index was calculated. To this end, TCR sequences were again rearranged into the ‘TCR_V_CDR3_TCR_J’ format and the absolute count of each TCR sequence per CD16^+^ CD8^+^ T_EMRA_ subtype was calculated. Morisita index was calculated using the *repOverlap* function implemented in *immunarch* package (0.7.0) with.*method = ‘morisita’* and.*col = ‘v+aa+j’*. Overlap was visualized using the *vis* function implemented in *immunarch*.

### Calculation of CDR3 abundance

To calculate the frequency of TRA and TRB CDR3 usage, TCR sequences were rearranged into the ‘TCR_V_CDR3_TCR_J’ format. The absolute count of each unique TCR sequence was calculated for each condition and for T cell receptor alpha and beta chains separately and divided by the total count of TCR sequences in the respective condition. The top 15 CDR3 sequences were visualized as bar charts.

### Analysis of flow cytometry data

To investigate the expression of surface protein receptors associated with CD8^+^ T cell exhaustion and to confirm the existence of CD16^+^ CD8^+^ T_EMRA_ subsets identified in the scRNA-seq dataset, we analyzed data from a publicly available flow cytometry dataset containing the same disease conditions as our dataset (44).

Flow cytometry data were processed using *FlowJo* version 10.8.1, and the output was subsequently analyzed and visualized in *R*.

### Statistical analysis

Statistical analyses were performed using *R* version 4.0.3 and *GraphPad Prism* version 9. Unless stated otherwise, significance was estimated with Wilcoxon rank sum tests. *p*-values were adjusted for multiple testing with the Benjamini-Hochberg method with FDR < 5%. Throughout the paper, significance was depicted as: **** = p-value ≤ 0.0001, *** = p-value ≤ 0.001, ** = p-value ≤ 0.01, * = p-value < 0.05.

## Results

### A population of exhausted CD8^+^ T cells is exclusive for severe COVID-19

**Figure 1:**
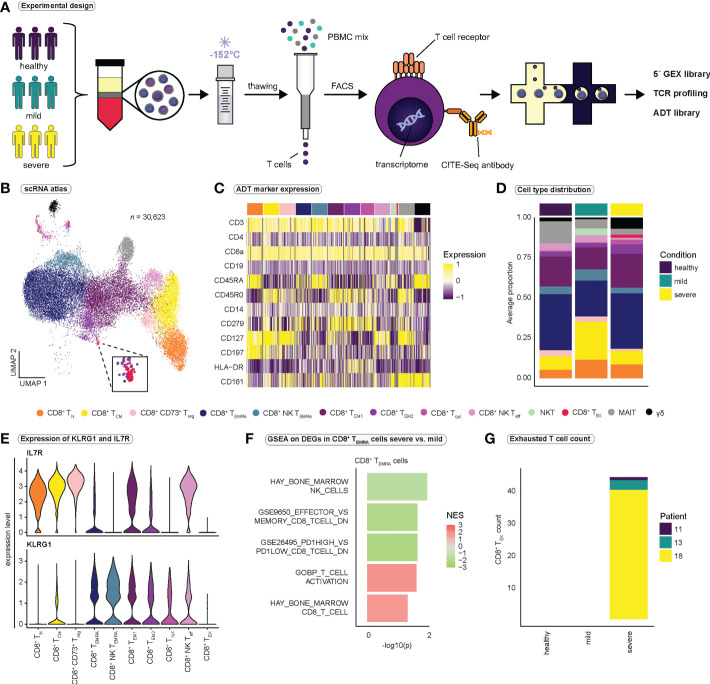
Study design and identification of functional subsets of CD8^+^ T cells. **(A)** Schematic overview of the study design. PBMCs were isolated from healthy volunteers, patients with mild, and patients with severe COVID-19. PBMCs were gradually frozen and stored at -152°C until further processing. After thawing, T cells were isolated using magnetic activated cell sorting. CD8^+^ T cells were obtained by FACS, and CD8^+^ T cells were then subjected to the 10x pipeline. **(B)** Integrated UMAP projection of all 13 CD8^+^ T cell subpopulations (*n* = 30,623) **(C)** Scaled expression of antibody-derived tag (ADT) markers (CITE-seq) per CD8^+^ T cell subpopulation. **(D)** Average proportion of CD8^+^ T cell subsets for the healthy (*n* = 2), mild (*n* = 6), and severe (*n* = 6) condition. Cell type proportions per patient are reported in **Figure S1C**. **(E)** Expression of *KLRG1* and *IL7R* per CD8^+^ T cell population. **(F)** Selected gene sets enriched in CD8^+^ T_EMRA_ cells when comparing severe to mild COVID-19 (for a full list of gene sets see **Table S4**) **(G)** Number of exhausted CD8^+^ T cells per condition and patient. FACS, fluorescence activated cell sorting; TCR, T cell receptor; GEX, gene expression; ADT, antibody-derived tag; CD8^+^ T_N_, CD8^+^ naïve T cells; CD8^+^ T_CM_, CD8^+^ central memory cells; CD8^+^ CD73^+^ Treg, CD8^+^ CD73^+^ regulatory T cells; CD8^+^ T_EMRA_, CD8^+^ terminally differentiated effector memory cells re-expressing CD45RA; CD8^+^ NK T_EMRA_, CD8^+^ NK-like terminally differentiated effector memory cells re-expressing CD45RA; CD8^+^ T_EM1_, CD8^+^ effector memory cells 1; CD8^+^ T_EM2_, CD8^+^ effector memory cells 2; CD8^+^ T_cyc_, CD8^+^ cycling effector cells; CD8^+^ NK T_eff_, CD8^+^ NK-like early effector T cells; NKT, atypical NKT cells; CD8^+^ T_EX_, CD8^+^ exhausted T cells; MAIT, Mucosal associated invariant T cells; γδ, γδ T cells; GSEA, gene set enrichment analysis.

To investigate CD8^+^ T cell heterogeneity in SARS-CoV-2 infection, we first performed single-cell immunoprofiling of CD8^+^ T cells (5´ sequencing, 10x Genomics) by combining scRNA-seq with TCR-seq and single-cell proteomics (CITE-seq antibodies, **Table S1**). To this end, CD8^+^ T cells were isolated from patients (**Table S2**) with mild COVID-19 (*n* = 6), severe COVID-19 (*n* = 6), and healthy controls (*n* = 3) and enriched for SARS-CoV-2-specific CD8^+^ T cells by FACS sorting using MHC I Dextramer reagents (**Figures 1A, S1A**). Of note, during the analysis, we detected a high proportion of a specific effector cell population in a healthy control who informed us of an unclear infection in early January 2020, approximately four months before the start of the study, and we therefore excluded this sample from the study (healthy controls, *n* = 2).

Unsupervised clustering and subsetting for CD8^+^ T cells captured 30,623 cells and 13 distinct clusters (**Figures 1B**, **S1B–J**). Functional annotation of T cell subclusters was based on RNA and protein expression of CD45RA, CCR7 (45) and specific T cell effector markers (**Figure 1C** and **Table S3**) (46). We identified 10 functional subpopulations of CD8^+^ T cells as well as three small populations that were annotated as atypical NKT cells (NKT), mucosal-associated invariant T cells (MAIT) and γδ T cells (**Figures 1B–D**, **S1B, C**). Since we aimed at investigating CD8^+^ αβ T cells, these three subpopulations were excluded from all further analysis. T cells expressing one or both of the T cell receptor genes characteristic of MAIT cells, TRAV1-2 and TRAJ33, were also excluded. Additionally, since we aimed to analyze clonal expansion, T cells with missing TCR-seq information (**Figure S1F**) were removed, leaving us with a final dataset of 25,506 cells for all downstream analyses.

Two clusters strongly expressed natural killer (NK) cell markers *KLRC2* and *NCR3*, and were therefore annotated as NK-like CD8^+^ early effector T cells (NK T_eff_) and NK-like CD8^+^ effector memory T cells re-expressing CD45RA (NK T_EMRA_). To validate our functional annotation, we investigated the expression of established markers *KLRG1* and *IL7R (* 47, 48) in each cluster (**Figure 1E**). In accordance with our annotation, terminally differentiated effector populations (CD8^+^ T_EMRA_ and CD8^+^ NK T_EMRA_) displayed high expression of *KLRG1* and low *IL7R* expression, while naïve (CD8^+^ T_N_) and central memory T cells (CD8^+^ T_CM_) expressed high levels of *IL7R* and low levels of *KLRG1* (**Figure 1E**). CD8^+^ NK T_eff_ cells and CD8^+^ effector memory T cells 1 (T_EM1_) expressed both *KLRG1* and high levels of *IL7R* and therefore may represent populations of memory precursor effector cells (MPEC) (47, 48).

To identify differences in CD8^+^ T cell populations between mild and severe COVID-19, we performed differential gene expression analysis between cells from mild and severe SARS-CoV-2 infection, followed by gene set enrichment analysis (GSEA) (**Figure 1F** and **Table S4**). GSEA revealed the enrichment of gene sets associated with T cell effector differentiation and T cell activation in severe COVID-19, whereas genes related to CD8^+^ T cell memory differentiation were enriched in mild COVID-19, indicating that CD8^+^ T cell fate is strongly directed toward a highly activated effector phenotype in severe SARS-CoV-2 infection (**Figure 1F** and **Table S4**). Furthermore, we observed enrichment of genes associated with the term ‘GSE26495_PD1HIGH_VS_PD1LOW_CD8_TCELL_DN’ in CD8^+^ T_EMRA_ cells in mild COVID-19. PD-1 is an inhibitory receptor that has been associated with T cell exhaustion, suggesting that CD8^+^ T_EMRA_ cells in mild SARS-CoV-2 infection, compared to severe infection, resemble cells with low PD-1 expression and thus exhibit a less exhausted phenotype (**Figure 1F**). Interestingly, we detected a small population of exhausted CD8^+^ T cells (T_EX_) characterized by expression of *TIGIT*, *CTLA4*, *CD279* (PD-1) and *HAVCR2* (Tim-3) that was exclusive to the severe COVID-19 group (**Figures 1B, G**), a finding which has been reported by others before (49, 50).

### Validation of CD8^+^ T cell exhaustion in COVID-19

**Figure 2:**
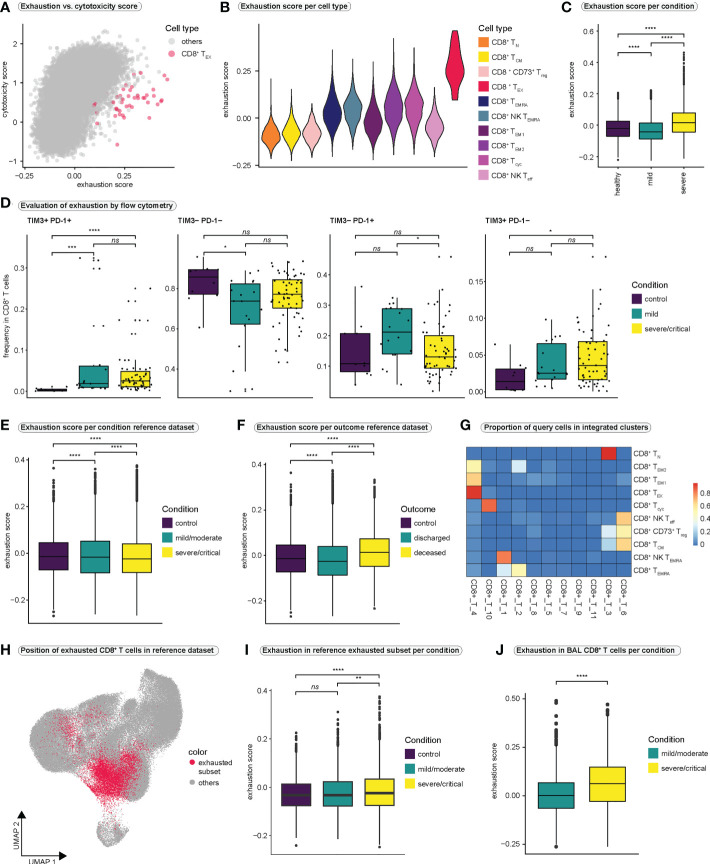
CD8^+^ T cell exhaustion in COVID-19 **(A)** Relationship between T cell exhaustion and cytotoxicity. Each CD8^+^ T cell is placed in the coordinate system according to its individual exhaustion and cytotoxicity score value. To highlight the position of CD8^+^ exhausted T cells, all other CD8^+^ T cell populations are colored in gray. A version of this plot with cell type-specific and condition-specific colors is available in **Figure S2A, S2B**, respectively. **(B)** Violin plot of exhaustion scores per cell type. **(C)** Exhaustion scores in all CD8^+^ T cells per condition. (Kruskal-Wallis test: *H (2*) = 2407, *p* < 0.0001; healthy vs. mild: *p* < 0.0001; healthy vs. severe: *p* < 0.0001, mild vs. severe: *p* < 0.0001) **(D)** Frequencies of cells expressing different combinations of the exhaustion markers PD-1 and TIM3 in all CD8^+^ T cells as examined by flow cytometry. (TIM3^+^PD-1^+^: Kruskal-Wallis test: *H*(2) = 21.59, *p* < 0.0001; control vs. mild: *p* = 0.0002; control vs. severe/critical: *p* < 0.0001, mild vs. severe/critical: *p* = 0.8933; TIM3^-^PD-1^-^: Kruskal-Wallis test: *H*(2) = 6.314, *p* = 0.0426; control vs. mild: *p* = 0.036; control vs. severe/critical: *p* = 0.1046, mild vs. severe/critical: *p* = 0.1689; TIM3^-^PD-1^+^: Kruskal-Wallis test: *H*(2) = 6.07, *p* = 0.0481; control vs. mild: *p* = 0.1303; control vs. severe/critical: *p* = 0.9359, mild vs. severe/critical: *p* = 0.0484, TIM3^+^PD-1^-^: Kruskal-Wallis test: *H*(2) = 6.287, *p* = 0.0431; control vs. mild: *p* = 0.1369; control vs. severe/critical: *p* = 0.0377, mild vs. severe/critical: *p* = 0.4863) **(E)** Exhaustion score in all CD8^+^ T cells in the reference dataset per condition. (Kruskal-Wallis test: *H*(2) = 149.7, *p* < 0.0001; control vs. mild: *p* < 0.0001; control vs. severe/critical: *p* < 0.0001, mild vs. severe/critical: *p* < 0.0001) **(F)** Exhaustion score in all CD8^+^ T cells in the reference dataset per outcome. (Kruskal-Wallis test: *H*(2) = 1328, *p* < 0.0001; control vs. discharged: *p* < 0.0001; control vs. deceased: *p* < 0.0001, discharged vs. deceased: *p* < 0.0001) **(G)** Heatmap displaying the proportions of query cell types that mapped to each cluster in the integrated dataset. High values indicate that most cells of a query cluster were assigned to a distinct cluster in the reference dataset. **(H)** Projection of cells that mapped to exhausted CD8^+^ T cells from the query dataset onto the UMAP of the reference dataset. **(I)** Exhaustion scores per condition in cells from the reference dataset that mapped to exhausted CD8^+^ T cells in the query dataset. (Kruskal-Wallis test: *H*(2) = 31.24, *p* < 0.0001; control vs. mild/moderate: *p* = 0.1054; control vs. severe/critical: *p* < 0.0001, mild/moderate vs. severe/critical: *p* = 0.0011) **(J)** Exhaustion scores per condition in CD8^+^ T cells from bronchoalveolar lavage fluid. (Kolmogorov-Smirnov test: *D* = 0.2422, *p* < 0.0001) BAL, bronchoalveolar lavage. **** = p-value ± 0.0001, *** = p-value ± 0.001, ** = p-value ± 0.01, * = p-value < 0.05, ns = not significant.

Exhausted T cells display a strong impairment of effector functions (51) and are typically seen in chronic infections or tumors, where chronic antigenic stimulation induces exhaustion (52). To investigate the functional relevance of exhaustion in our dataset, we calculated cytotoxicity and exhaustion scores previously described (32, 53) (**Figures 2A**, **S2A, B**). Exhaustion appeared to increase almost linearly with increasing cytotoxicity, resulting in high cytotoxicity and exhaustion scores in particular effector cell populations (**Figure S2A**). T_EX_ cells exhibited extremely high exhaustion scores with presumably moderate cytotoxicity (**Figures 2A**, **S2A**), suggesting a disturbed balance between the expression of inhibitory receptors and the cytotoxic functions of CD8^+^ T cells. Comparison of exhaustion scores between the CD8^+^ T cell populations revealed highest exhaustion scores in T_EX_ cells (**Figures 2B**, **S2C**). Comparison of exhaustion scores of all CD8^+^ T cells between conditions revealed significantly higher exhaustion scores in severe SARS-CoV-2 infection compared to mild disease or healthy controls (**Figure 2C**).

To further investigate CD8^+^ T cell exhaustion, we first analyzed a publicly available flow cytometry dataset (**Figures 2D**, **S2D**) generated by the COMBAT consortium (44). Analysis of PD-1 and TIM3 expression, two markers of T cell exhaustion, revealed significantly higher frequencies of double-positive cells (PD-1^+^TIM3^+^) in COVID-19 compared to healthy controls, although no significant difference was observed between mild and severe infection (**Figure 2D**).

Since the size of our single-cell dataset was limited, we wanted to investigate exhaustion in a large public dataset by Ren et al. (34). Therefore, after filtering the reference dataset for 5´-sequencing samples and samples derived from frozen PBMCs to match our samples, we extracted all CD8^+^ T cells, resulting in a reference dataset of 114,209 CD8^+^ T cells. However, by investigating exhaustion scores in the reference dataset, we were unable to clearly identify a CD8^+^ T cell population characterized by extremely high exhaustion scores (**Figure S2E**). In addition, comparison of exhaustion scores of all CD8^+^ T cells between conditions revealed lower exhaustion scores in severely/critically ill patients compared to mild/moderate illness or controls (**Figure 2E**). Interestingly, when comparing exhaustion scores in all CD8^+^ T cells between outcome groups, patients who succumbed to COVID-19 displayed the highest exhaustion scores (**Figure 2F**).

To uncover exhausted T cells in the reference dataset, we integrated the reference dataset with our CD8^+^ T cell dataset. Most of our T_EX_ cells mapped onto one distinct subcluster (cluster CD8^+^_T_4) in the integrated dataset (**Figure 2G**). Projecting all cells from this subcluster back to the UMAP of the CD8^+^ T cell reference dataset revealed that the exhausted T cells were distributed across the UMAP space of the reference dataset, rather than forming a distinct cluster (**Figure 2H**). This explained why we were unable to identify a distinct cluster with a high exhaustion score in the reference dataset. Thus, we focused on the newly identified subset of exhausted T cells in the reference dataset and compared exhaustion scores within these cells between conditions. We observed significantly higher exhaustion scores in the exhausted subset in severe/critical COVID-19 as compared to mild/moderate disease and healthy controls (**Figure 2I**). Similarly, patients who died due to COVID-19 revealed highest exhaustion scores in their exhausted subset (**Figure S2F**).

Lastly, we asked whether exhaustion is more pronounced in CD8^+^ T cells that are close to the site of infection. To this end, we generated a second CD8^+^ T cell reference dataset by filtering all CD8^+^ T cells from the Ren dataset (34) for cells derived from bronchoalveolar lavage (BAL) fluid. Also in this dataset we did not identify a cluster characterized by excessively high exhaustion scores (**Figure S2G**). However, when comparing the exhaustion scores of all BAL-derived CD8^+^ T cells between the conditions and the outcomes of SARS-CoV-2 infection, the group of severely infected individuals and the group that succumbed to COVID-19 clearly displayed higher exhaustion scores than the group with mild infection or the group that recovered from SARS-CoV-2 infection, respectively (**Figures 2J** and **S2H**). Since the differences in exhaustion scores between the conditions were more pronounced in BAL-derived CD8^+^ T cells, it is conceivable that CD8^+^ T cells that are close to the site of infection undergo more severe exhaustion, an effect that seems to be pronounced in severe and critical SARS-CoV-2 infection. For an overview over the reference and the integrated datasets, we refer to **Figures S3A-J**.

### SARS-CoV-2 infection drives terminal effector differentiation towards an NK-like phenotype

**Figure 3:**
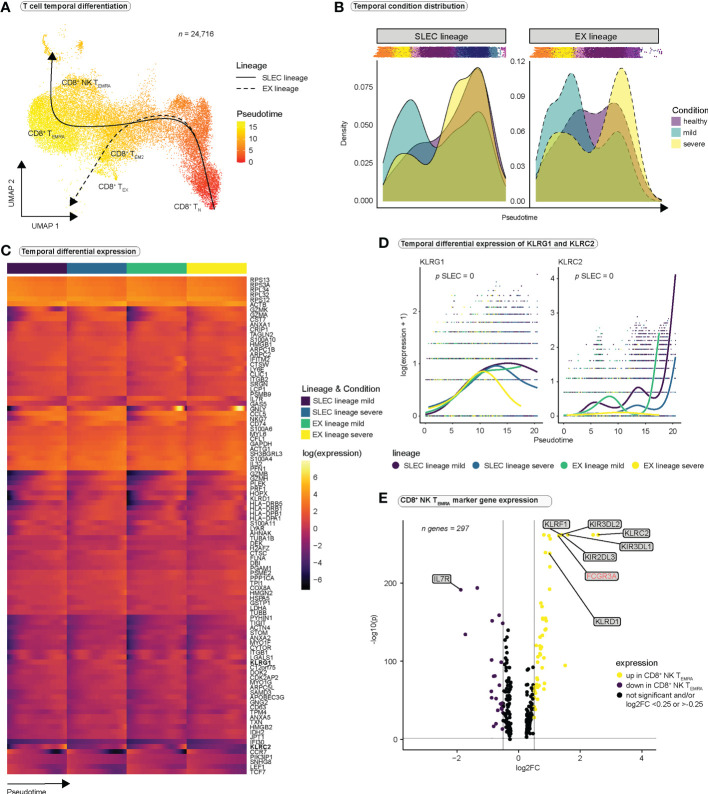
CD8^+^ T cells differentiate towards an NK-like phenotype during SARS-CoV-2 infection. **(A)** Pseudotimes and estimated trajectories projected onto the integrated UMAP of cell types likely originating in naïve CD8^+^ T cells (*n* = 24,716). For trajectory inference with Slingshot, naïve CD8^+^ T cells were manually chosen as the origin of differentiation. **(B)** Temporal distribution of cell densities for all three conditions across pseudotime. Shifts in distribution between the mild and the severe condition for the short-lived effector cells (SLEC) and exhaustion (EX) lineage were tested with the Kolmogorov-Smirnov method (EX: *D* = 0.40237, *p* < 2.2e-16, SLEC: *D* = 0.31004, *p* < 2.2e-16). **(C)** Heatmap depicts differentially expressed genes between the progenitor and differentiated cell populations across pseudotime (start vs. end testing). **(D)** Smoothed expression of *KLRG1* and *KLRC2* across pseudotime with the y-axis on natural logarithmic scale. *p*-values report the result of differential expression analysis between progenitor and differentiated cell states across pseudotime (start vs. end testing). An extended panel of genes and their UMAP projections are reported in **Figure S4**. **(E)** Volcano plot of genes differentially expressed in CD8^+^ NK-like T_EMRA_ cells.

To investigate CD8^+^ T cell differentiation we predicted pseudotime trajectories using Slingshot (41). As input for inference of differentiation trajectories by Slingshot, we manually defined the cluster of CD8^+^ naïve T cells as the root of all differentiation trajectories. Slingshot predicted two differentiation pathways (**Figures 3A**, **S4A**), both originating in naïve CD8^+^ T cells and passing through the effector memory stage. Trajectory 1 continued toward the T_EMRA_ and the NK T_EMRA_ stages and was termed ‘short lived effector cell lineage’ (SLEC). Trajectory 2 ended in close proximity to the exhausted CD8^+^ T cell population; we therefore termed this lineage ‘exhaustion lineage’ (EX) (**Figure 3A**). Investigation of pseudotime-dependent cell densities revealed highest cell densities in early stages of pseudotime in mild COVID-19 for both lineages, whereas there appeared to be a significant shift in cell densities toward the late stages of differentiation in severe COVID-19 (**Figure 3B**). Surprisingly, cells from healthy controls showed a similar distribution of cell densities as cells from severely infected patients. Because **Figure 3B** displays only the number of cells at a given differentiation stage, and bias may occur in single-cell experiments regarding the ratio of different cell populations to each other, comparisons of absolute frequencies in single-cell datasets should be evaluated with caution. In our case, it is conceivable that the distribution in healthy controls is partly due to the smaller number of cells in this group.

To verify our lineage assignments and to compare trajectories between mild and severe disease conditions, we performed trajectory-based differential expression analysis for sequencing data (tradeSeq) (42) (**Table S5**). tradeSeq revealed a significant downregulation of *IL7R* (**Figure S4B**) and a significant upregulation of *KLRG1* (**Figures 3C, D**) along the SLEC lineage, suggestive of effector differentiation, while significant upregulation of *CTLA4* along the EX lineage mainly in the severe condition (**Figure S4B**), supported the concept of an ‘exhaustion lineage’. Comparison between the disease conditions indicated significantly stronger expression of certain NK cell receptors (*CD160*, *KLRC2*, *KLRC3*, *KLRF1*, *KIR3DL2*, *NCR1*, *NCR3*) along the SLEC lineage in mild COVID-19 compared to severe COVID-19 (**Figures 3D**, **S4C, D**). These results suggest that NK cell receptors are expressed during effector differentiation in SARS-CoV-2 infections and that in severe COVID-19 the expression profile of these receptors differs from the one in mild COVID-19. Since CD8^+^ NK T_EMRA_ cells represent a final state of the SLEC lineage (**Figure 3A**), and this population is particularly characterized by the expression of NK cell receptors, we concluded that the observed differences could be the result of differences in the differentiation of CD8^+^ NK T_EMRA_ cells in severe SARS-CoV-2 infection. To deeply characterize this population, we examined the genes differentially expressed in CD8^+^ NK T_EMRA_ cells as compared to all other CD8^+^ T cell populations (**Figure 3E** and **Table S3**). Strikingly, CD8^+^ NK T_EMRA_ cells displayed strong upregulation of *FCGR3A* (CD16), *KLRF1* and certain killer cell immunoglobulin-like receptors (KIRs), strong downregulation of *IL7R* (**Figure 3E**), and expression of *IKZF2*. CD16^+^ CD8^+^ T cell populations have been previously observed in different chronic viral infections (54, 55). Naluyima et al. have described a population of terminally differentiated CD16^+^ CD8^+^ T cells characterized by the expression of KIRs, KLRF1, IKZF2 as well as low expression levels of IL7R that were able to mediate antibody-dependent cellular cytotoxicity (ADCC) (54). These cells strongly resembled our CD8^+^ NK T_EMRA_ population and we verified low IL7R (CD127) expression at a surface protein level using our CITE-seq data (**Figure S4E**). In summary, SARS-CoV-2 infection seems to induce a population of terminally differentiated CD16^+^ CD8^+^ effector T cells, which are observed in other chronic viral infections and appear to differ between the disease conditions.

### T cell receptor diversity in mild and severe COVID-19

**Figure 4:**
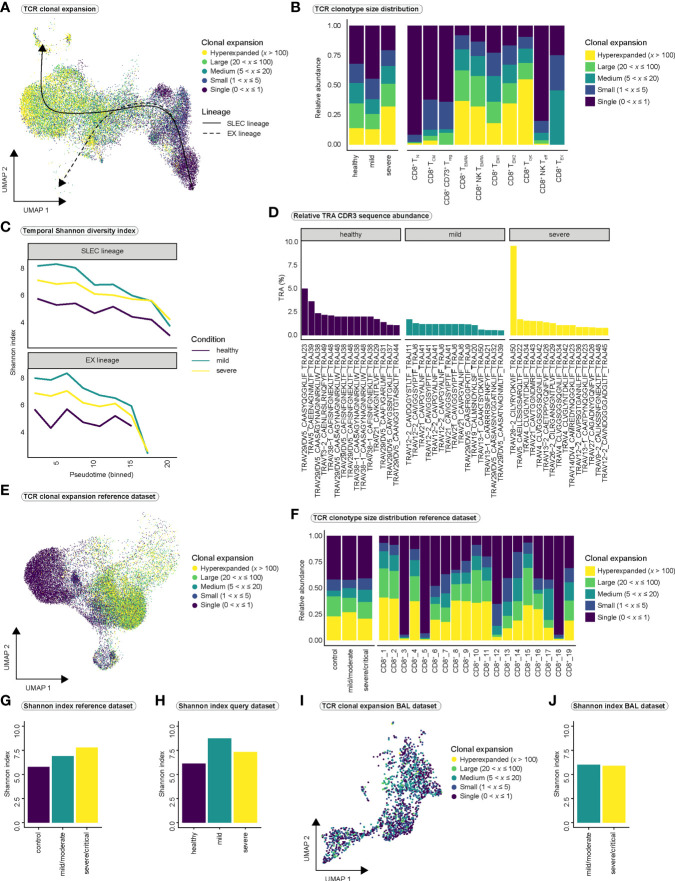
Clonal expansion and TCR diversity in COVID-19. **(A)** T cell receptor clonal expansion projected onto the integrated UMAP of cell types. **(B)** Distribution of clonal expansion within the conditions (left) and within the CD8^+^ T cell populations (right), displayed as relative abundance of clonotype expansion groupings (**Figure S5A** is referred to for abundance per cell type within each condition). **(C)** Shannon diversity index as a measure of clonal diversity across pseudotime for the three conditions among SLEC and EX lineages. Shannon index was calculated on the whole TCR sequences, including TRA and TRB chains. **(D)** Relative proportion of CDR3 sequences of the 15 most abundant clones to the total number of CDR3 sequences per condition for the TRA chain (**Figure S5C** is referred to for the relative proportion of CDR3 sequences for the TRB chain). **(E)** TCR clonal expansion projected onto the UMAP of the PBMC-derived CD8^+^ reference dataset. **(F)** Distribution of clonal expansion within the conditions (left) and within the CD8^+^ T cell populations (right), displayed as relative abundance of clonotype expansion groupings for the PBMC-derived reference dataset. (**Figure S5D** is referred to for abundance per cell type within each condition of the reference dataset). **(G)** Shannon diversity index per condition for the PBMC-derived reference dataset and **(H)** for our query dataset. **(I)** TCR clonal expansion projected onto the UMAP of the BAL-derived CD8^+^ reference dataset. **(J)** Shannon diversity index per condition for the BAL-derived reference dataset. TCR, T cell receptor; SLEC, short-lived effector cell; EX, exhaustion; TRA, T cell receptor alpha chain; TRB, T cell receptor beta chain; CDR3, Complementarity determining region 3; BAL, bronchoalveolar lavage.

**Figure 5:**
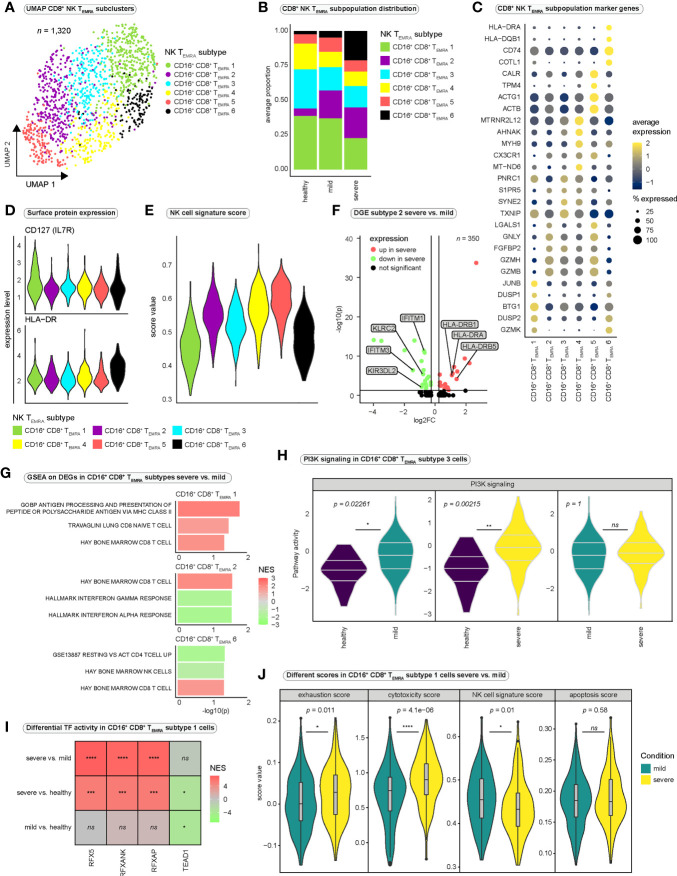
Heterogeneity among CD8^+^ NK-like terminally differentiated effector memory T cells re-expressing CD45RA. **(A)** Integrated UMAP projection of subclustered CD8^+^ NK-like T_EMRA_ cells (CD16^+^ CD8^+^ T_EMRA_ cells) (*n* = 1,320). **(B)** Average proportion of CD16^+^ CD8^+^ T_EMRA_ subsets for the three conditions. Cell type proportions per patient are reported in **Figure S6A**. **(C)** Average expression of marker genes differentially expressed in the six CD16^+^ CD8^+^ T_EMRA_ subsets. **(D)** Surface expression (CITE-seq) of IL7R and HLA-DR per CD16^+^ CD8^+^ T_EMRA_ subtype. **(E)** NK cell signature scores of the six CD16^+^ CD8^+^ T_EMRA_ subsets. **(F)** Differentially expressed genes in CD16^+^ CD8^+^ T_EMRA_-2 cells between the severe and the mild disease condition. **(G)** Selected significantly enriched gene sets for genes differentially expressed in the indicated CD16^+^ CD8^+^ T_EMRA_ subtypes between mild and severe disease groups. Positive normalized enrichment scores (NES) indicate enrichment in the severe disease condition. **(H)** PI3K pathway activity in CD16^+^ CD8^+^ T_EMRA_-3 cells estimated with PROGENy. Significance was tested using Wilcoxon rank sum test. All PROGENy pathways are reported in **Figure S6G**. **(I)** Differential transcription factor activity (DoRothEA) estimated with *msviper* in CD16^+^ CD8^+^ T_EMRA_-1 cells between the severe and the mild condition. Positive NES values indicate increased activity in severe SARS-CoV-2 infection. **(J)** Different functional scores applied to CD16^+^ CD8^+^ T_EMRA_-1 cells and compared between severe and mild COVID-19 (Wilcoxon rank sum test). DGE, differential gene expression; log2FC, log2 fold change; GSEA, gene set enrichment analysis; NES, normalized enrichment score; PI3K, Phosphoinositide 3-kinase; TF, transcription factor.

Next, we asked whether there are differences in clonal expansion of CD8^+^ T cells between the COVID-19 disease conditions. Superimposing TCR clonality on our UMAP indicated an increase in clonal expansion along the differentiation trajectories (**Figure 4A**), consistent with clonal expansion and differentiation after antigen encounter. As expected, effector cell populations (CD8^+^ T_EM1_, CD8^+^ T_EM2_, CD8^+^ T_cyc_, CD8^+^ T_EMRA_, CD8^+^ NK T_EMRA_) displayed highest relative abundance of hyperexpanded clones and hyperexpansion appeared to be more pronounced in severe COVID-19 (**Figures 4B**, **S5A**). Only CD8^+^ NK T_EMRA_ cells appeared to be more expanded in mild than in severe COVID-19 (**Figure S5A**). Because strong expansion of individual clones can affect TCR diversity, we calculated the Shannon diversity index as a measure of TCR repertoire diversity over pseudotime using the whole TCR sequence and observed a decrease in diversity for all three conditions (**Figure 4C**). However, at most time points, the TCR repertoire appeared to be more diverse in mild SARS-CoV2 infection than in severe infection in both lineages (**Figure 4C**). Analysis of TCR overlap for both the T cell receptor alpha (TRA) and T cell receptor beta (TRB) chains revealed no significant differences in overlap between the two disease states and the diseased and healthy states (**Figure S5B**). Next, we calculated the relative contribution of the CDR3 sequences of the top 15 clonotypes to the overall CDR3 pool in the respective conditions for the TRA chain (**Figure 4D**) and the TRB chain (**Figure S5C**). Even though we observed one clone that was highly hyperexpanded and occupied a larger portion of the TCR space than all other clones in severe COVID-19, we did not observe major differences between mild and severe disease (**Figures 5D**, **S5C**). The Shannon diversity index and analysis of CDR3 abundance both indicated lowest TCR diversity in healthy controls.

We validated our findings in the large PBMC-derived CD8^+^ T cell reference dataset (**Figures 4E–H**) but observed only minor differences in the distribution of clonotype sizes between conditions (**Figures 4F**, **S5D**). As in our dataset, control CD8^+^ T cells displayed the lowest TCR diversity as measured by Shannon index in the reference dataset (**Figure 4G–H**). However, in contrast to our data (**Figures 4C, H**), mildly infected individuals did not show the highest TCR diversity (**Figures 4G–H**). In summary, we observe highly expanded CD8^+^ effector T cell populations in SARS-CoV-2 infection. However, our results do not suggest major differences in T cell expansion or T cell receptor diversity between mild and severe COVID-19. TCR diversity seems to increase in response to SARS-CoV-2 infection as indicated by the higher Shannon index in mild and severe COVID-19 compared to healthy controls. It is conceivable that more T cells are released into the blood during the course of infection to diversify the TCR repertoire and to thus induce an immune response against a maximum number of viral antigens. This may explain the observed differences in Shannon index between healthy controls and disease conditions.

Finally, we investigated clonal expansion in the BAL-derived CD8^+^ reference dataset (**Figures 4I, J**, **S5E**). This analysis confirmed our previous observation that no major differences in overall TCR diversity exist between mild and severe SARS-CoV-2 infection (**Figure 4J**). However, we observed a difference in clonal expansion between mild and severe infection in a cell type we termed BAL_CD8^+^_7. While this cell type exhibited the lowest expansion in severe COVID-19, it showed strong clonal expansion in mild COVID-19 (**Figure S5E**). Interestingly, BAL_CD8^+^_7 cells were mainly characterized by the expression of *FCGR3A* and *KLRF1* (**Figure S5F**), suggesting that this population resembles our CD8^+^ NK-like T_EMRA_ cells. Since we also observed stronger expansion of this cell type in mild than in severe SARS-CoV-2 infection in our dataset (**Figure S5A**), the findings of the BAL dataset confirmed our previous findings.

### Subtypes of NK cell-like CD8^+^ T cells in SARS-CoV-2 infection

Trajectory analysis suggested a CD16^+^ CD8^+^ T cell population (previously termed CD8^+^ NK T_EMRA_ cells) as a final state of CD8^+^ terminal effector differentiation that seemed to differ in phenotypic characteristics between mild and severe COVID-19. Recently, Georg et al. discovered highly activated and highly cytotoxic CD16^+^ T cells among the CD4^+^ and CD8^+^ T cell compartments which were more abundant in severe COVID-19 as compared to mild disease or healthy controls and had the ability to elicit antibody-dependent cellular cytotoxicity (ADCC) (56). Because of the potential relevance of these CD16^+^ CD8^+^ T cells for both, effective antiviral response as well as immunopathology, we focused our analysis on CD8^+^ NK T_EMRA_ cells.

To investigate cellular heterogeneity among CD16^+^ CD8^+^ T cells, we subclustered our CD8^+^ NK T_EMRA_ population, resolving six subclusters (**Figure 5A**), which we termed CD16^+^ CD8^+^ T_EMRA_ cells-1 to 6. All subpopulations were present in every condition (**Figure 5B**) and in most patients (**Figure S6A**). However, the CD16^+^ CD8^+^ T_EMRA_-1 cluster accounted for the largest subpopulation and appeared to be more abundant in mild disease and healthy controls, whereas the CD16^+^ CD8^+^ T_EMRA_-6 cluster was more abundant in severe disease (**Figure 5B**). Analysis of clonal expansion revealed stronger clonal expansion in CD16^+^ CD8^+^ T_EMRA_ cells in mild disease, while the lowest proportion of hyperexpanded clones was observed in subcluster 6 cells (**Figure S6B**). To quantify overlap in TCR repertoires between the CD16^+^ CD8^+^ T_EMRA_ subsets, we calculated the Morisita index (**Figure S6C**). While there appeared to be large overlap in TCR repertoire between subsets 1-5, CD16^+^ CD8^+^ T_EMRA_-6 cells displayed lowest levels of overlap with other subclusters (**Figure S6C**). The strong similarity in TCR repertoires of subsets 1-5 may indicate that these subpopulations are derived from each other in a continuous differentiation process, whereas subset 6 cells may arise as a result of an alternative differentiation process. Differential gene expression analysis between the 6 subclusters (**Table S6**) revealed high expression of *BTG1* in CD16^+^ CD8^+^ T_EMRA_-1 cells (**Figure 5C**), a gene that has been shown to be involved in the maintenance of a quiescent state in T cells (57). In contrast, CD16^+^ CD8^+^ T_EMRA_-6 cells differentially expressed MHC class II genes *HLA-DRA* and *HLA-DQB1* (**Figure 5C**), which are known to be upregulated by activated T cells (58, 59), indicating a high activation state of subset 6 cells, similar to the populations described by Georg et al. (56). We further investigated expression of specific genes related to NK cell function and observed strong expression of *KLRF1* and *FCGR3A* in CD16^+^ CD8^+^ T_EMRA_-2 to 5 cells (**Figure S6D**), indicating that these cells strongly resembled the population described by Naluyima et al. in HIV infection (54). Despite their effector phenotype, CD16^+^ CD8^+^ T_EMRA_-1 cells exhibited expression of *IL7R* (**Figure S6D**), which may indicate a more naïve phenotype, compared to the other subpopulations, or the potential to develop into memory T cells (48, 60). Surface expression of IL7R (CD127) on subcluster 1 cells and surface expression of HLA-DR on subcluster 6 cells were validated using our CITE-seq data (**Figure 5D**). Interestingly, we further observed expression of CD161, another NK cell receptor on subcluster 2-5 cells (**Figure S6E**).

To quantify and compare functional characteristics of the different subpopulations, we calculated functional scores for every cell and compared it between the subsets (**Figures 5E**, **S6F**). Interestingly, CD16^+^ CD8^+^ T_EMRA_-1 and -6 cells displayed the lowest NK cell signature score, suggesting differences in NK-like differentiation between the subpopulations (**Figure 5E**). Regarding the expression of genes involved in T cell quiescence and homeostasis, it is conceivable that subcluster 1 cells represent a CD16^+^ CD8^+^ progenitor population from which, controlled by the influences of the prevailing milieu, the various other subpopulations emerge, acquiring further NK-like characteristics during differentiation.

We next performed differential gene expression analysis between the conditions for every CD16^+^ CD8^+^ T_EMRA_ subcluster (**Figure 5F** and **Table S6**). Despite that only a limited number of differentially expressed genes proved significant due to the small sample size, the differential expression of MHC class II genes in severe SARS-CoV-2 infection in different subpopulations (**Figure 5F** and **Table S6**) supported the suggestion of a strong activation state of NK-like CD8^+^ T cells in severe COVID-19. Differential expression of NK cell receptor genes, such as *KLRC2* and *KIR3DL2* in CD16^+^ CD8^+^ T_EMRA_-2 cells in mild COVID-19 compared with severe COVID-19 further strengthened the hypothesis of profound differences in NK-like differentiation between disease conditions.

Performing GSEA on the differentially expressed genes revealed the enrichment of the gene ontology (GO-) term “GOBP Antigen Processing And Presentation Of Peptide Or Polysaccharide Antigen *Via* MHC Class II” in subcluster 1 in severe disease (**Figure 5G** and **Table S7**), which is consistent with the differential expression of MHC class II genes (**Table S6**). Strikingly, while various CD8^+^ T cell signature genes were enriched in different CD16^+^ CD8^+^ T_EMRA_ subtypes in severe SARS-CoV-2 infection, cells in subcluster 6 were negatively enriched in genes associated with “Hay Bone Marrow NK cells” (**Figure 5G** and **Table S7**). Thus, we concluded that compared to CD16^+^ CD8^+^ T_EMRA_ cells from patients with mild SARS-CoV-2 infection, cells from severely affected individuals are impaired in their NK-like differentiation and are rather characterized by CD8^+^ T cell-like traits, than by NK-like characteristics (**Figure 5G**).

We then inferred pathway activity in CD16^+^ CD8^+^ T_EMRA_ cells and observed significantly stronger PI3K signaling in CD16^+^ CD8^+^ T_EMRA_-3 cells in the diseased conditions when compared to healthy controls (**Figures 5H, S6G**). Several NK cell receptors mediate their signals *via* the PI3K pathway (61, 62) and especially ADCC, a key function of NK cells, which is triggered upon CD16 ligation is mediated *via* the PI3K pathway (63). Thus, increased PI3K activity could indicate increased ligation of Fcγ-receptor-IIIa and a relevance for ADCC in the immune response to COVID-19. Inference of transcription factor activity predicted significantly stronger activity of the Regulatory Factor X (RFX) transcription factor family in severe COVID-19 when compared to mild disease in all 6 NK-like subtypes (**Figures 5I**, **S6H, I**; **Table S8**). Since these transcription factors are involved in the regulation of MHC class II genes (64), higher activity is in accordance with differential expression of MHC class II genes and the enrichment in GO-terms related to antigen processing *via* MHC class II in severe SARS-CoV-2 infection.

To further dissect functional differences between mild and severe SARS-CoV-2 infection, we focused on CD16^+^ CD8^+^ T_EMRA_-1 cells, which we suspected to be the earliest of the six subpopulations in terms of NK-like differentiation. We assigned different functional scores to all CD16^+^ CD8^+^ T_EMRA_-1 cells and compared them between mild and severe disease. While there were no significant differences in apoptosis (apoptosis score), CD16^+^ CD8^+^ T_EMRA_-1 cells were significantly more exhausted and cytotoxic in severe COVID-19 (**Figure 5J**). Furthermore, a significantly higher NK cell signature score in mild COVID-19 (**Figure 5J**) further supported the hypothesis of impaired NK-like differentiation in severe SARS-CoV-2 infection, which appeared to be present already at the early stage of CD16^+^ CD8^+^ T_EMRA_-1 cells.

In summary we observed several subsets of CD16^+^ CD8^+^ T_EMRA_ cells in SARS-CoV-2 infection, which rather represent a continuum in NK-like differentiation than distinct cellular subpopulations. Four subsets strongly expressed *KLRF1* and *FCGR3A*, displayed surface expression of CD161 and resembled a population of CD16^+^ CD8^+^ T cells that has previously been described in other viral infections (54, 55). Another subset with the lowest amount of hyperexpanded clones, the lowest overlap in TCR repertoire with other subsets, and surface expression of HLA-DR, indicating high activation status, was more abundant in severe COVID-19. A larger subset of CD16^+^ CD8^+^ T cells that displayed surface expression of IL7R and a lower NK cell signature than the previously mentioned subsets was present in all three conditions. However, cells in this subset differed strongly between mild and severe disease. Investigation of NK cell characteristics revealed profound differences between mild and severe COVID-19 and suggested impaired NK-like differentiation in severe COVID-19. It is conceivable that CD16^+^ CD8^+^ T_EMRA_-1 cells represent the initial population from which, depending on the cytokine milieu and the stimulation of specific surface receptors, the different CD16^+^ subpopulations develop.

### CD16^+^ CD8^+^ NK-like derived gene sets are related to severity and outcome of SARS-CoV-2 infection

Since our CD16^+^ CD8^+^ T cell subset only consisted of 1,320 cells, we aimed at verifying the existence of an NK-like CD8^+^ T cell population by flow cytometry as well as in the CD8^+^ reference dataset (34).

**Figure 6:**
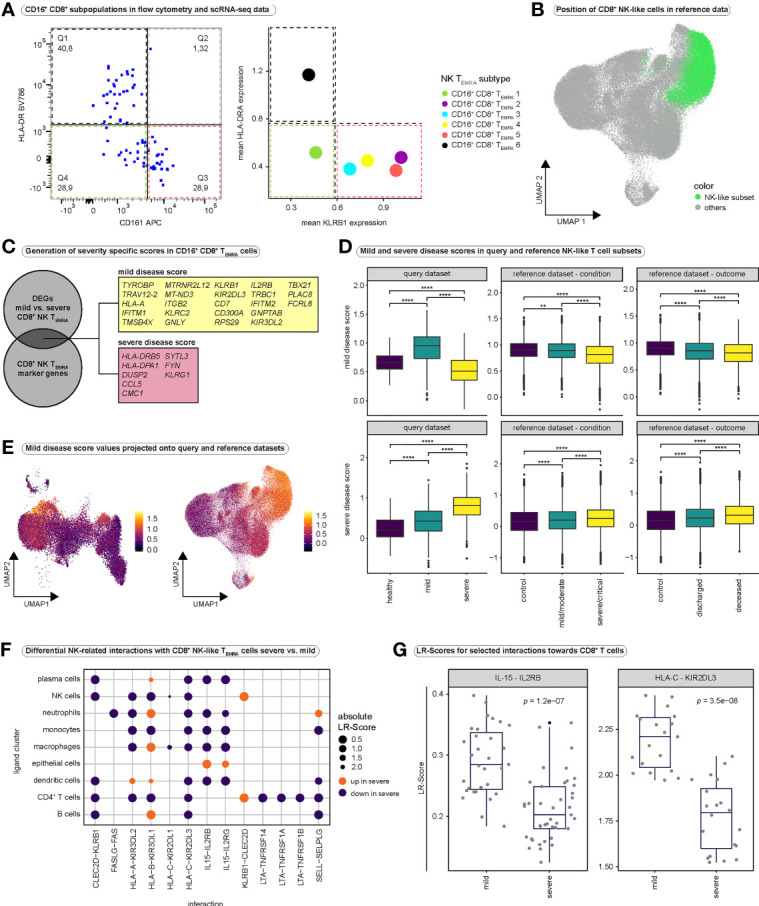
Validation of CD16^+^ CD8^+^ T_EMRA_ cells and cell-cell interaction analysis. **(A)** Validation of the existence of CD16^+^ CD8^+^ T_EMRA_ subsets, identified in scRNA-seq data. The expression of HLA-DR (highly expressed in CD16^+^ CD8^+^ T_EMRA_-6 cells) and CD161 (highly expressed by CD16^+^ CD8^+^ subsets 2 to 5) was investigated in single, live CD16^+^ CD8^+^ T cells in a flow cytometry dataset (left panel). As a comparison, the mean expression of the corresponding genes (*HLA-DRA* as an example of an HLA-DR gene, as well as *KLRB1* encoding CD161) in the CD16^+^ CD8^+^ subpopulations is shown (right panel). **(B)** UMAP embedding of the CD8^+^ T cell reference dataset (*n* = 114,209). Cells that mapped together with the CD8^+^ NK-like T_EMRA_ cells from our query dataset after integration are highlighted in green. **(C)** Schematic illustrating the generation of mild and severe disease scores. Differential gene expression analysis was performed between mild and severe disease groups for all CD16^+^ CD8^+^ T_EMRA_ cells. Differentially expressed genes that overlapped with highly significant marker genes of CD16^+^ CD8^+^ T_EMRA_ cells (CD8^+^ NK-like T_EMRA_ cells) were identified. Genes that were differentially upregulated in cells from mild disease (average log2-fold change > 0.25) were combined into the mild disease score, whereas genes that were differentially downregulated in cells from mild disease (average log2-fold change < 0.25) were combined into the severe disease score. **(D)** Comparison of mild (top) and severe (bottom) disease scores in NK-like CD8^+^ T cell subsets between conditions in our query dataset (left), between conditions in the PBMC-derived reference dataset (middle) and between outcome groups in the PBMC-derived reference dataset (right). Kruskal-Wallis test was used for significance testing. (query mild disease score: Kruskal-Wallis test: *H*(2) = 462.6, *p* < 0.0001; healthy vs. mild: *p* < 0.0001; healthy vs. severe: *p* < 0.0001, mild vs. severe: *p* < 0.0001; query severe disease score: Kruskal-Wallis test: *H*(2) = 330.5, *p* < 0.0001; healthy vs. mild: *p* < 0.0001; healthy vs. severe: *p* < 0.0001, mild vs. severe: *p* < 0.0001; reference mild disease score condition: Kruskal-Wallis test: *H*(2) = 778.6, *p* < 0.0001; control vs. mild/moderate: *p* = 0.0046; control vs. severe/critical: *p* < 0.0001, mild/moderate vs. severe/critical: *p* < 0.0001; reference severe disease score condition: Kruskal-Wallis test: *H*(2) = 246.2, *p* < 0.0001; control vs. mild/moderate: *p* < 0.0001; control vs. severe/critical: *p* < 0.0001, mild/moderate vs. severe/critical: *p* < 0.0001; reference mild disease score outcome: Kruskal-Wallis test: *H*(2) = 258.2, *p* < 0.0001; control vs. deceased: *p* = < 0.0001; control vs. discharged: *p* < 0.0001, deceased vs. discharged: *p* < 0.0001; reference severe disease score outcome: Kruskal-Wallis test: *H*(2) = 203.9, *p* < 0.0001; control vs. deceased: *p* = < 0.0001; control vs. discharged: *p* < 0.0001, deceased vs. discharged: *p* < 0.0001) **(E)** Mild disease score values projected onto the UMAP embeddings of our query CD8^+^ T cell dataset (left) and the reference CD8^+^ T cell dataset (right). Projections for severe disease score values are depicted in **Figure S7G**. **(F)** Differential ligand-receptor interactions between severe and mild COVID-19. To assess interactions between CD8^+^ T cells and non-T cells our dataset was integrated with the whole reference dataset and interactions were predicted using *LIANA*. Differential interactions were then calculated using *CrossTalkeR* for selected interactions, relevant in NK cell development and function. A group of ligand clusters was selected and NK-like CD8^+^ T_EMRA_ cells were regarded as receptor cluster. The size of the dots indicates the absolute value of the differential LR-Score. The color indicates the direction of the change in ligand-receptor interactions; orange indicates increased interactions in severe COVID-19, purple indicates decreased interactions in severe COVID-19. **(G)** Boxplots displaying differences in LR-Scores for selected interactions between severe and mild COVID-19. All CD8^+^ T cell populations were regarded as receptor population and all other populations (except megakaryocytes) were regarded as ligand population for this purpose. DEGs, differentially expressed genes; LR-Score = ligand-receptor score.

To this end, we again used the public flow cytometry dataset (44) (**Figure S7A**). CD16^+^ CD8^+^ T_EMRA_ subsets were combined into three groups, based on the expression of HLA-DR and CD161. CD16^+^ CD8^+^ T_EMRA_-1 cells only displayed low expression of *HLA-DRA* and *KLRB1* (encoding CD161) in the scRNA-seq data (CD161^-^ HLA-DR^-^), whereas subsets 2-5 expressed high levels of *KLRB1* (CD161^+^ HLA-DR^-^), and CD16^+^ CD8^+^ T_EMRA_-6 cells displayed high expression of *HLA-DRA* (CD161^-^ HLA-DR^+^) (**Figure 6A**). Flow cytometry identified all three subsets of CD16^+^ CD8^+^ T cells (**Figure 6A**). Even though CD161^-^ HLA-DR^+^ (CD16^+^ CD8^+^ T_EMRA_-6 cells) were least frequent in both datasets, flow cytometry did not show higher frequencies of this population in severely and critically ill patients (**Figure S7B**). Additionally, while in flow cytometry data CD161^-^ HLA-DR^-^ cells (CD16^+^ CD8^+^ T_EMRA_-1 cells) accounted for the majority of CD16^+^ CD8^+^ T cells, in scRNA-seq data subsets 2-5 (CD161^+^ HLA-DR^-^) made up the largest proportion of CD16^+^ CD8^+^ T cells (**Figure S7B**).

Next, we aimed at identifying CD16^+^ CD8^+^ T cells in the large PBMC-derived CD8^+^ reference dataset. Most of our CD8^+^ NK-like T_EMRA_ cells mapped onto one distinct subcluster (cluster CD8^+^_T_1) in the integrated dataset (**Figure 2G**). Projecting all cells from this subcluster to the UMAP of the CD8^+^ T cell reference dataset revealed that the NK-like CD8^+^ T cell population represented a distinct region in the UMAP space of the reference dataset (**Figure 6B**). We verified expression of *FCGR3A* in these reference NK-like CD8^+^ T cells and observed strong expression of *FCGR3A* (**Figure S7C**).

Next, we investigated whether genes that characterize CD16^+^ CD8^+^ T_EMRA_ cells have a relevance for disease severity. We therefore performed differential gene expression analysis between CD16^+^ CD8^+^ T_EMRA_ cells from mild and severe COVID-19 (**Table S9**). Among the differentially expressed genes, we identified several genes that overlapped with highly significant marker genes of our CD8^+^ NK-like T_EMRA_ population (**Table S3**). We combined marker genes that were differentially upregulated in mild COVID-19 into a ‘mild disease score’ and marker genes differentially upregulated in severe disease into a ‘severe disease score’(**Figure 6C**). Indeed, patients with mild COVID-19 displayed significantly higher “mild disease score” values, while severely affected patients displayed highest “severe disease score” values in their CD8^+^ NK-like T cells (**Figure 6D**). To independently validate our scores, we subsetted all cells from the reference dataset that mapped together with our CD8^+^ NK-like T_EMRA_ cells in the integrated dataset (**Figures S7D–F**) and calculated score values for these reference cells. Also in the reference data, control subjects and patients with mild/moderate SARS-CoV2 infection displayed significantly higher “mild disease score” values and patients with severe infection displayed highest “severe disease score” values (**Figure 6D**). Strikingly, patients that succumbed to COVID-19 displayed lowest mild disease score, and highest severe disease score values as compared to patients who survived and healthy controls (**Figure 6D**). However, it is important to note, that due to the nature of single-cell experiments and the comparison of thousands of cells, even minor changes can yield significant p-values.

Mapping these disease scores back to our whole dataset as well as the whole CD8^+^ reference dataset revealed high specificity of the mild disease score to CD8^+^ NK-like cells, while the severe disease score did not show specificity to a certain population (**Figures 6E**, **S7G–I**). In summary, our molecular signature that was specific to CD16^+^ CD8^+^ T cells was significantly elevated in CD16^+^ CD8^+^ T cells from individuals with mild COVID-19, while the molecular signature that was elevated in patients with severe SARS-CoV-2 infection did not show the same specificity for CD16^+^ CD8^+^ T cells. Thus, we conclude that a proper and specific NK-like differentiation of CD8^+^ effector T cells might be protective against severe COVID-19, while a dysfunctional NK-like differentiation with nonspecific changes such as hyperactivation in NK-like CD8^+^ T cells might be a mechanism involved in the pathogenesis of severe COVID-19.

### Differential cell-cell interactions could drive differences in NK-like CD8^+^ T cell differentiation

To investigate factors that could drive NK-like differentiation of CD8^+^ T cells, we integrated our dataset with selected samples (**Table S10**) from the same publicly available single-cell dataset as described above (34) and performed cell-cell interaction analysis. We first focused our analysis on NK cell-related interactions between non-CD8^+^ populations and NK-like T_EMRA_ cells (**Figures 6F**, **S8A, B**) and investigated differences in these interactions between mild and severe SARS-CoV-2 infection (**Figure 6F**). Differential interaction analysis (**Table S11**) revealed differences in various interactions that have been related to NK cell development (65). Particularly, interactions between ligands on non-CD8^+^ populations and KIRs on NK-like T_EMRA_ cells seemed to be more abundant in mild COVID-19 (**Figures 6F**, **S8A, C**). Moreover, we observed increased *IL15* interactions in mild disease (**Figure 6F**). IL15 has been shown to be a highly relevant cytokine in the development of NK cells (66–68). Next, we compared predicted ligand-receptor (LR) scores for selected interactions, this time considering all CD8^+^ T cell populations as receptor populations (**Figure 6G**, **S8C**). Interestingly, LR-Scores for *IL15*-*IL2RB* interactions were significantly higher in mild COVID-19 compared to severe COVID-19 (**Figure 6G**). Moreover, LR-Scores for various interactions between MHC-I molecules and NK cell receptors were significantly higher in mild SARS-CoV-2 infection (**Figures 6G**, **S8C**). KIR2DL1, KIR2DL3, KIR3DL1 and KIR3DL2 (**Figures 6G**, **S8C**) are inhibitory NK cell receptors and enhanced interactions *via* these receptors on CD8^+^ T cells could indicate a more balanced functional regulation of CD8^+^ T cells in mild COVID-19. In contrast, we observed significantly increased LR-Scores for various CC chemokine-interactions in severe COVID-19 compared to mild COVID-19 (**Figure S8C**). In summary, IL15 could be involved in the acquisition of an NK-like phenotype during CD8^+^ effector differentiation in SARS-CoV-2 infection. Differences in NK cell-related interactions as well as differences in cytokine milieu could drive the observed differences in CD16^+^ CD8^+^ T_EMRA_ cells between mild and severe disease.

## Discussion

In this study we explored cellular heterogeneity within the CD8^+^ T cell compartment in SARS-CoV-2 infection and investigated differences between mild and severe COVID-19. We observed large heterogeneity among CD8^+^ T cells, representing different functional subsets. A subpopulation of exhausted CD8^+^ T cells was observed in severe COVID-19 in our scRNA-seq dataset. However, whereas flow cytometry indicated increased exhaustion in COVID-19, we did not observe significant differences between mild and severe infection. Targeted analysis of exhausted T cells in a large reference dataset ultimately revealed significantly higher exhaustion scores in patients with severe SARS-CoV-2 infection, as well as in patients who died due to COVID-19. These differences were even more pronounced in lung-derived CD8^+^ T cells, suggesting that the location of CD8^+^ T cells is relevant for the occurrence of CD8^+^ T cell exhaustion in COVID-19. Our findings are in line with previous reports about an increase in exhaustion characteristics in CD8^+^ T cells in severe SARS-CoV-2 infection (50, 69, 70). Trajectory-analysis indicated two differentiation pathways, one corresponding to short-lived effector cell differentiation and one corresponding to T cell exhaustion. Upregulation of NK cell-related genes along the SLEC lineage pointed us to a subset of terminally differentiated CD8^+^ effector T cells that were characterized by the expression of *FCGR3A* (encoding CD16), *IKZF2* (encoding the transcription factor Helios) and *KLRF1* (encoding the NK cell receptor NKp80). Deeper investigation of this NK-like CD8^+^ T_EMRA_ population revealed various CD16^+^ CD8^+^ T cell subsets.

A first subset (CD16^+^ CD8^+^ T_EMRA_-1) was present in all three conditions and was characterized by moderate expression of *FCGR3A*, surface expression of IL7R as well as a relatively low NK-like phenotype. Besides this first CD16^+^ CD8^+^ T_EMRA_ population, four strongly clonally expanded subsets (CD16^+^ CD8^+^ T_EMRA_-2 to -5), characterized by high expression of *FCGR3A*, *KLRF1* and surface expression of CD161 were observed in all conditions, whereas a highly activated and less expanded subset (CD16^+^ CD8^+^ T_EMRA_-6) was enriched in severe cases of COVID-19.

Differential gene expression analysis suggested substantial differences in activation status and expression of NK cell receptors in CD16^+^ CD8^+^ T_EMRA_ cells between mild and severe disease and applying functional scores to CD16^+^ CD8^+^ T_EMRA_-1 cells revealed higher cytotoxicity, a higher exhaustion signature as well as decreased NK cell signature scores in severe SARS-CoV-2 infection as compared to mild.

We confirmed the existence of different CD16^+^ CD8^+^ T cell subsets by flow cytometry, although the relative proportions of the different subsets in all CD16^+^ CD8^+^ T cells differed between flow cytometry and scRNA-seq. These differences could be explained by two crucial points. First, there is a discrepancy between gene expression and protein expression, such that a high abundance of mRNA of a gene does not translate 1:1 into a high expression of the corresponding protein. Because the CD16^+^ CD8^+^ subpopulations in the scRNA-seq dataset are defined by their transcriptome, whereas in flow cytometry they are defined at the protein level, this may lead to shifts in the frequency distribution of the subpopulations. Second, however, scRNA-seq is much better suited to characterize subtle differences between cell populations, whereas the discrimination of subtypes in flow cytometry was based on two markers only.

CD16^+^ CD8^+^ T cell subsets have been observed in various conditions. In chronic, untreated HIV infection a CD16^+^ CD8^+^ population with high expression of NKp80 and Helios transcription factor has been described (54). Previously, Björkström et al. had detected a similar, and clonally expanded subpopulation in chronic hepatitis C virus (HCV) infection (55). Both papers reported the ability of CD16^+^ CD8^+^ T cells to mediate ADCC or at least effector functions in response to engagement of CD16 (54, 55). These populations strongly resembled our CD16^+^ CD8^+^ T_EMRA_-2 to -5 populations, suggesting a potential relevance for ADCC in the antiviral response to SARS-CoV-2 infection. Moreover, CD16^+^ CD8^+^ T_EMRA_-2 to -5 cells displayed strong surface expression of CD161. CD161 is an NK cell receptor that has been suggested to mark long-lived antigen-specific T cells within the CD4^+^ compartment (71). Additionally, CD161^+^ T cells have been reported to respond to IL-12 and IL-18 in a TCR-independent manner (72). We therefore hypothesize that properly differentiated ‘bona fide’ CD16^+^ CD8^+^ T cells also contribute to antiviral defense by TCR-independent mechanisms through their NK-like differentiation. Of particular interest is the fact that ADCC can also be mediated by non-neutralizing antibodies, which may have particular relevance for the design of future vaccines (73, 74).

On the other hand, a highly activated population of CD16^+^ CD8^+^ T_EMRA_ cells has recently been described in severe COVID-19 and the authors suggested a role for these cells in immunopathology of severe infection by ADCC-mediated endothelial damage (56). The existence of pathogenic T cell populations has already been suggested by others in COVID-19 (14) and other diseases (75, 76). Interestingly, the highly activated phenotype and the specificity to the severe disease condition reported by Georg et al. resembled the CD16^+^ CD8^+^ T_EMRA_ subset 6 in our dataset. We therefore hypothesize that CD16^+^ CD8^+^ T cells are not inherently pathological in nature. We propose a model in which terminally differentiated CD16^+^ CD8^+^ T cells develop in the context of SARS-CoV-2 infection as part of effector differentiation, but in which differences in further differentiation of these cells eventually occur depending on the surrounding cytokine milieu, as well as stimulation of specific surface receptors. Adequate differentiation conditions the emergence of the ‘bona fide’ *IZKF2* ^+^ *KLRF1* ^+^ CD16^+^ CD8^+^ T cells, which may contribute to the clearance of the virus through ADCC and thus protect against severe courses. Defective NK-like differentiation could lead to the emergence of the highly activated and potentially pathogenic CD16^+^ CD8^+^ subtypes. It is conceivable that these dysfunctional subpopulations contribute to disease progression as suggested by Georg et al. (56). This is supported by the observation that a gene set, differentially expressed in NK-like CD8^+^ T_EMRA_ cells in mild COVID-19 when compared to severe disease, is highly specific to NK-like CD8^+^ T_EMRA_ cells, whereas a gene set differentially expressed in NK-like CD8^+^ T_EMRA_ cells in severe disease does not show high specificity to this population. Thus, high expression of NK-like CD8^+^ T_EMRA_-specific genes is related to mild disease while unspecific changes in CD16^+^ CD8^+^ T cells, especially high activation state, seems to be related to severe disease.

Indeed, a recent single-cell analysis identified CD16^+^ CD8^+^ T cells in smokers and non-smokers (77). When compared to non-smokers, CD16^+^ CD8^+^ T cells in smokers displayed elevated *TOX* expression, a transcription factor involved in T cell exhaustion (78), indicating a dysfunctional state of these cells (77). Further, expression of certain cytotoxic effector molecules as well as certain MHC class II genes was elevated in CD16^+^ CD8^+^ T cells from smokers (77). These results clearly illustrate that under certain circumstances, CD16^+^ CD8^+^ T cells can acquire dysfunctional phenotypes. Interestingly, Georg et al. showed that C3a is able to induce differentiation of CD16^+^ CD8^+^ T cells (56). To gain further insights into factors that might drive the differentiation of CD16^+^ CD8^+^ T cells, we performed cell-cell interaction analysis. When comparing predicted interactions with CD8^+^ T cells between mild and severe SARS-CoV-2 infection, we observed stronger interactions with KIR receptors (KIR3DL2, KIR2DL1, KIR2DL3) in CD16^+^ CD8^+^ T_EMRA_ cells derived from mildly affected individuals. It is known that inhibitory KIRs counteract activating signals downstream of the TCR (79). Additionally, in transgenic mice, ligation of KIR2DL3 by its cognate MHC class I ligand has been shown to reduce activation-induced cell death, thereby promoting survival of a subset of CD8^+^ memory cells (80). These findings may partially explain the differences in activation status between CD16^+^ CD8^+^ T_EMRA_ cells in mild and severe disease. Furthermore, filtering our cell-cell interaction data for mechanisms involved in NK-cell differentiation revealed increased IL-15 signaling towards CD8^+^ T cells in mild COVID-19 (**Figure 6G**). Previous studies have demonstrated the ability of IL-15 to induce an NK-like phenotype in CD8^+^ T cells (81, 82), suggesting differences in IL-15 signaling between mild and severe SARS-CoV-2 infection as a potential mechanism that drives differences in the generation of different CD8^+^ NK-like T cell phenotypes.

Nonetheless, our study has some limitations. A main limitation of our study is the small number of healthy controls, which limits the interpretation of comparisons with the healthy control group. To overcome this limitation, we validated our main observations and key points in a large reference dataset. Another limitation is the heterogeneity of diseased patients with respect to the duration of infection at the time point of sampling, a limitation that is also present in the reference dataset and that must be taken into account when interpreting the results. Finally, when interpreting statistical comparisons in single-cell datasets, it must be considered that due to the comparison of thousands of single cells, even smaller changes can cause statistical significance.

In summary, by investigating CD8^+^ T cells in SARS-CoV-2 infection at high resolution, we observed different subsets of NK-like CD16^+^ CD8^+^ T_EMRA_ cells, a population that only few reports have observed before. We deeply characterized these cells and suggest a role for CD16^+^ CD8^+^ T cells in the immune response against SARS-CoV-2 by mediating ADCC. By inferring cell-cell interactions, we identify factors that could be involved in the differentiation of CD16^+^ CD8^+^ T cells. We suggest that differences in the cytokine milieu in severe as compared to mild SARS-CoV-2 infection result in disturbances of this NK-like differentiation process, potentially leading to the generation of dysfunctional or even pathogenic CD16^+^ CD8^+^ T cell subsets that are characterized by high activation status and low NK-like phenotype, whereas properly differentiated NK-like T_EMRA_ cells that acquire NK cell specific characteristics confer protection against the virus. Although we validated our results in a flow cytometry and in a large scRNA-seq dataset, future studies will have to validate our findings in larger cohorts to dissect the factors that drive NK-like differentiation of CD8^+^ T cells as well as their role in health and disease. However, the ability to elicit ADCC represents another mechanism for combating the virus and could also represent a crucial role for non-neutralizing antibodies in viral defense, which would also affect future vaccine designs.

## Data availability statement

A list of CITE-seq antibodies used in this study is provided in Table S1. Code used for data analysis is available at https://github.com/KramannLab/Covid_immunoprofiling. Raw sequencing data are available at the European Nucleotide Archive (ENA) at: https://www.ebi.ac.uk/ena/browser/view/PRJEB57300. Pre-processed data including R objects are available at Zenodo: 10.5281/zenodo.7129229

## Ethics statement

The studies involving human participants were reviewed and approved by Ethical Board of the RWTH Aachen University Hospital. The patients/participants provided their written informed consent to participate in this study.

## Author contributions

Conceptualization: FS, CKup, SH, RK. Data Curation: FS, MH, HK, SH. Formal Analysis: FS, MH, HK, TB, FT, EF, JN, TA, SH. Funding Acquisition: EF, IC, RK. Investigation: FS, MH, MB, NT, PZ, VB, FW. Methodology: FS, MH, HK, CKup, SH, RK. Project Administration: FS, SH, RK. Resources: IK, JK, DF, TK, JE, PB, CKur, GM, NM, MD, RS, JS-R, IC, SH, RK. Software: FS, MH, HK, TB, FT, EF, JN, JS-R, IC, SH. Supervision: IC, SH, RK. Validation: FS, MH. Visualization: FS, MH, HK, TB, JN, SH. Writing – Original Draft Preparation: FS, MH. Writing – Review & Editing: HK, TB, FT, EF, JN, MB, NT, Ckup, IK, JK, DF, TA, PZ, TK, JE, VB, FW, PB, Ckur, GM, NM, MD, RS, JS-R, IC, SH, RK. All authors contributed to the article and approved the submitted version.

## Supplementary material

Supplementary Table 3 Marker genes differentially expressed in each CD8^+^ T cell subtype.

Supplementary Table 4 Results of differential gene expression analysis and gene set enrichment analysis on genes differentially expressed in CD8^+^ T cell populations between mild and severe COVID-19

Supplementary Table 5 tradeSeq results.

Supplementary Table 6 Marker genes differentially expressed in CD16^+^ CD8^+^ T cell subtypes and differential gene expression analysis results in CD16^+^ CD8^+^ T cell subtypes between the conditions.

Supplementary Table 7 Results of gene set enrichment analysis on genes differentially expressed in CD16^+^ CD8^+^ T cell subtypes between the conditions.

Supplementary Table 8 Results of differential transcription factor activity in CD16^+^ CD8^+^ T cell subtypes between the conditions.

Supplementary Table 9 Differential gene expression analysis results between mild and severe SARS-CoV-2 infection in CD8^+^ NK-like T_EMRA_ cells.

Supplementary Table 11 Cell-cell interactions analysis results.
