# Supplementary material for: Dissecting CD8+ T cell pathology of severe SARS-CoV-2 infection by single-cell immunoprofiling
Source: Front Immunol. 2022 Dec 16;13:1066176. doi: 10.3389/fimmu.2022.1066176 (PMC9800604; doi:10.3389/fimmu.2022.1066176)
Supplement: Supplementary file 1 [file DataSheet_1.pdf]

Supplementary Material

1 Supplementary Tables and Figures

1.1 Supplementary Figures

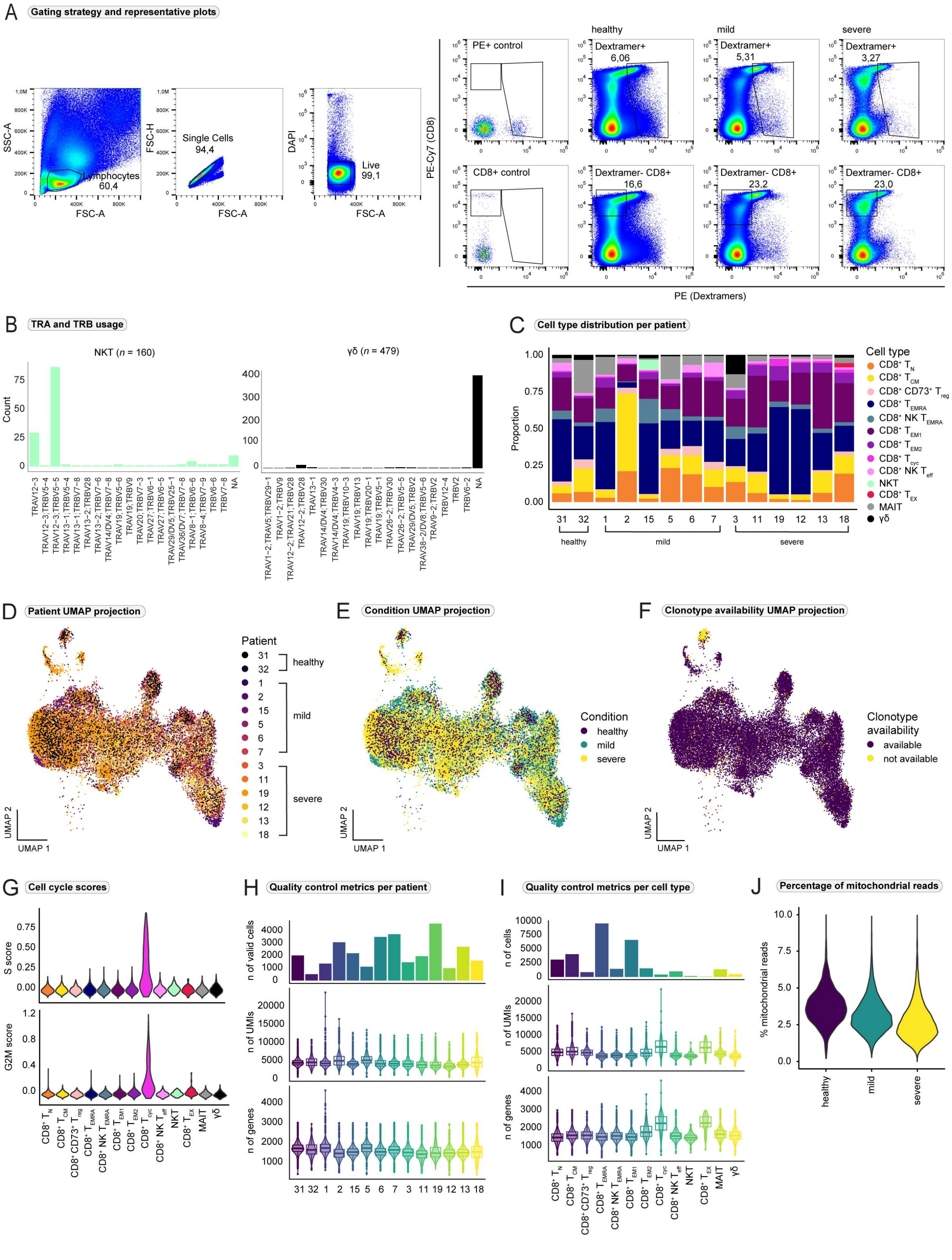

Figure S1. Additional information on the generation of single-cell data. (A) FACS gating strategy. Two different populations were sorted for, a PE^+ (Dextramer reagent-positive) and a $CD8^+$ PE^- population. **(B)** T cell receptor alpha (TRA) and T cell receptor beta chain (TRB) usage in atypical NKT cells and γδ T cells. **(C)** Distribution of $CD8^+$ T cell subsets per patient. **(D)** Per patient-origin and **(E)** per condition-origin projected onto the integrated UMAP. **(F)** Clonotype availability per cell projected onto the integrated UMAP. **(G)** Cell cycle scores per $CD8^+$ T cell subtype. **(H)** Overall single-cell RNA-seq data quality control metrics per patient. **(I)** Overall single-cell RNA-seq data quality control metrics per cell type. **(J)** Percentage of mitochondrial read content per condition. PE, phycoerythrin; UMI, unique molecular identifier.

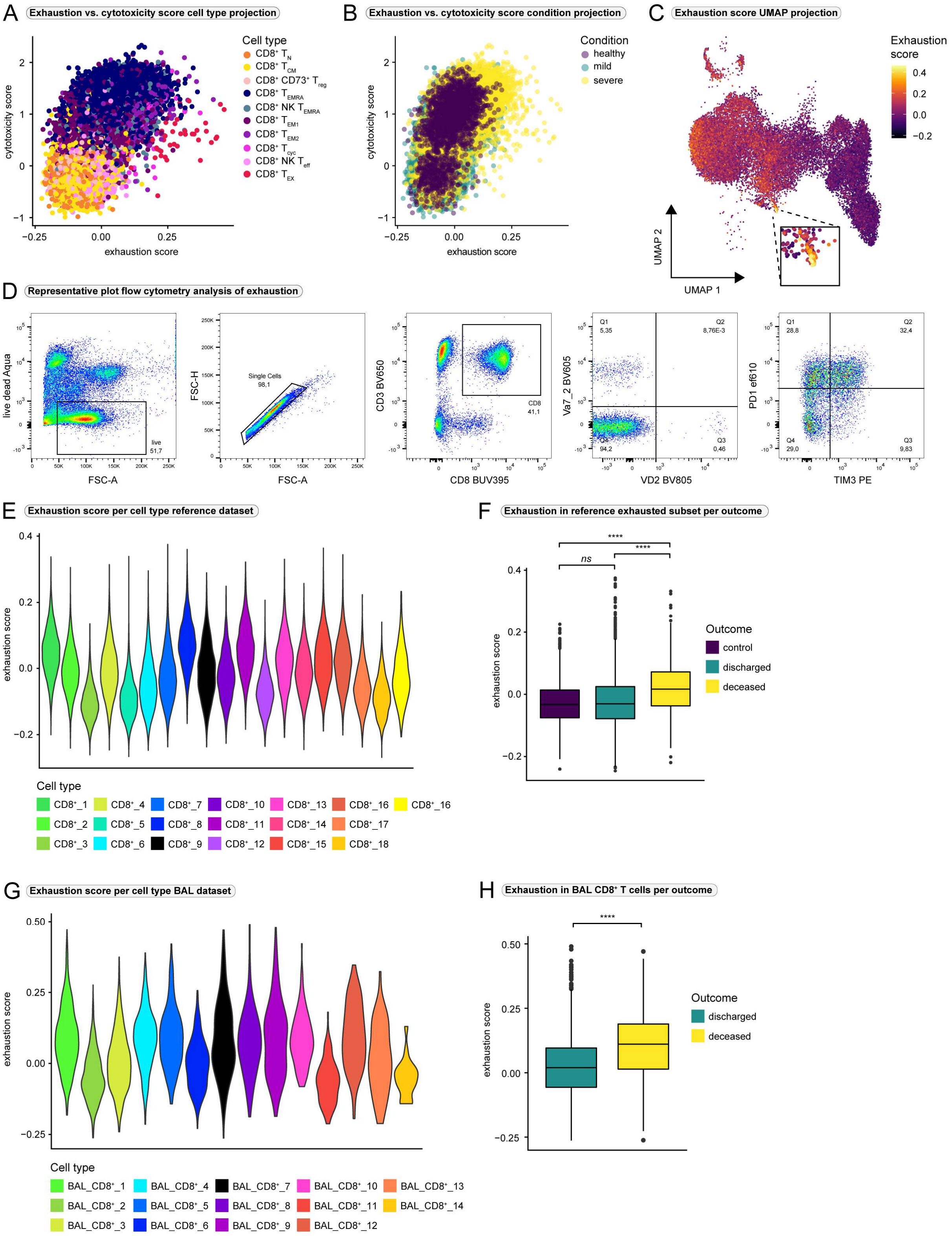

Figure S2. CD8$^+$ T cell exhaustion in COVID-19. (A) Cell type projection of the relationship between T cell exhaustion and cytotoxicity. Each single CD8$^+$ T cell is placed in the coordinate system according to its individual exhaustion and cytotoxicity score value with cell type-specific colors. **(B)** Condition projection of the relationship between T cell exhaustion and cytotoxicity. **(C)** UMAP projection of exhaustion scores with a magnification of the area where

the exhausted T cells are located. **(D)** Representative gating strategy of the flow cytometry analysis of exhaustion in CD8$^+$ T cells. After exclusion of dead cells and doublets, we first gated on CD8$^+$ T cells. By gating on TCR Vδ2$^-$ and TCR Vα7.2$^-$ cells we excluded major populations of $\gamma\delta$ T cells and MAIT cells. Finally, we quantified the frequency of cells expressing different combinations of the exhaustion markers PD-1 and TIM3. **(E)** Violin plot of the exhaustion score per cell type in the PBMC-derived reference dataset. **(F)** Exhaustion scores per outcome in cells from the reference dataset that mapped to exhausted CD8$^+$ T cells in the query dataset. (Kruskal-Wallis test: $H(2) = 245.9$, $p < 0.0001$; control vs. discharged: $p = 0.0525$; control vs. deceased: $p < 0.0001$, discharged vs. deceased: $p < 0.0001$) **(G)** Violin plot of the exhaustion score per cell type in the BAL-derived reference dataset. **(H)** Exhaustion scores per outcome in CD8$^+$ T cells from bronchoalveolar lavage fluid. (Kolmogorov-Smirnov test: $D = 0.3033$, $p < 0.0001$) BAL, bronchoalveolar lavage.

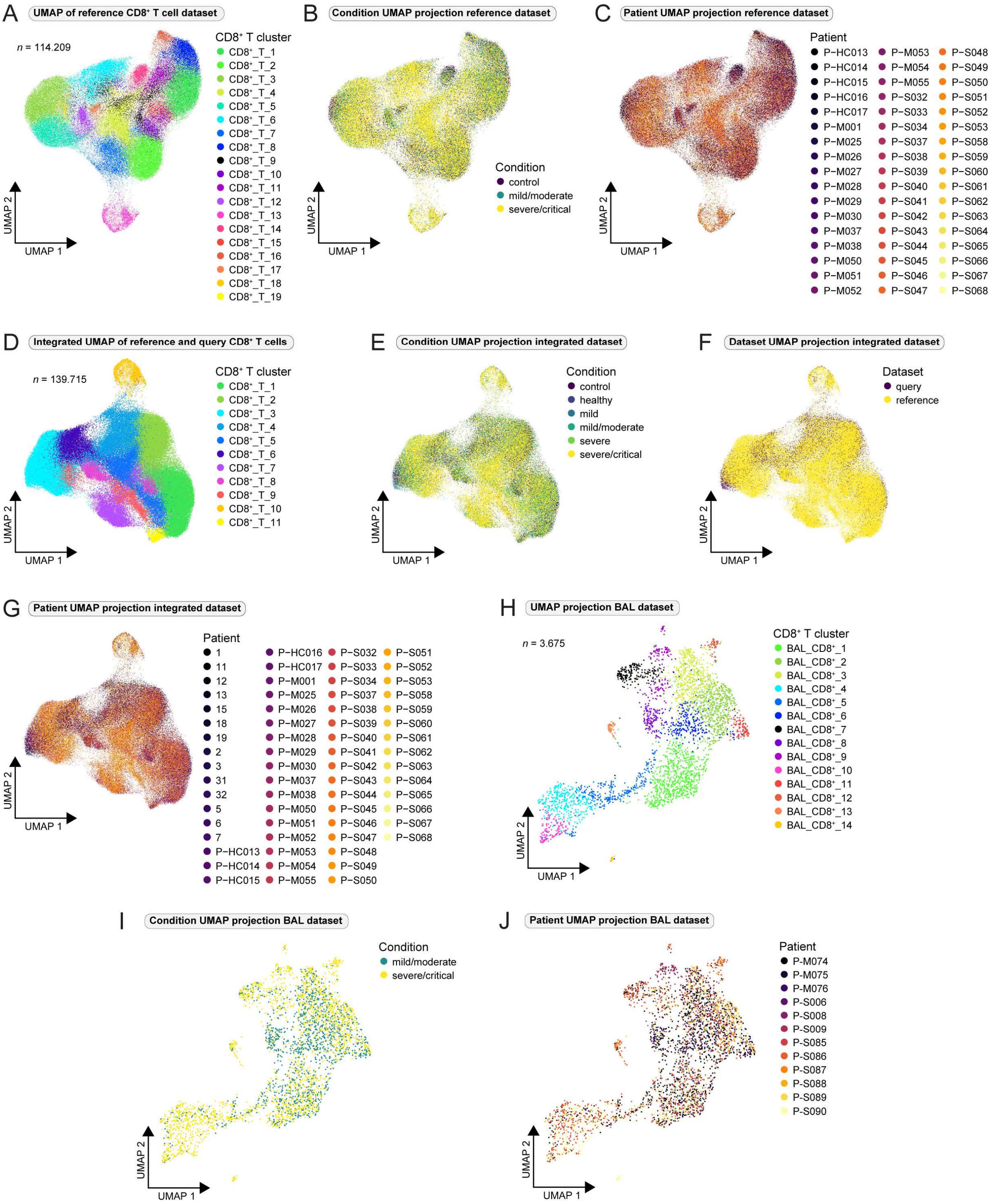

Figure S3. UMAP embeddings of reference and integrated datasets. (A) UMAP embedding of the PBMC-derived reference CD8$^+$ T cell dataset (n = 114,209). Reference mapping was performed using *Seurat v4*. **(B)** Per condition-origin and **(C)** per patient-origin projected onto the UMAP of the PBMC-derived reference CD8$^+$ T cell dataset. **(D)** UMAP embedding of the integrated CD8$^+$ T cell dataset (our query dataset with the PBMC-derived reference dataset, n = 139.715). **(E)** Per condition-origin, **(F)** per dataset-origin and **(G)** per patient-origin projected onto the UMAP of the integrated query and reference CD8$^+$ T cell datasets. **(H)** UMAP embedding of the BAL-derived CD8$^+$ T cell reference dataset (n = 3.675). **(I)** Per condition-

origin and **(J)** per patient-origin projected onto the UMAP of the BAL-derived reference $CD8^+$ T cell dataset. BAL, bronchoalveolar lavage.

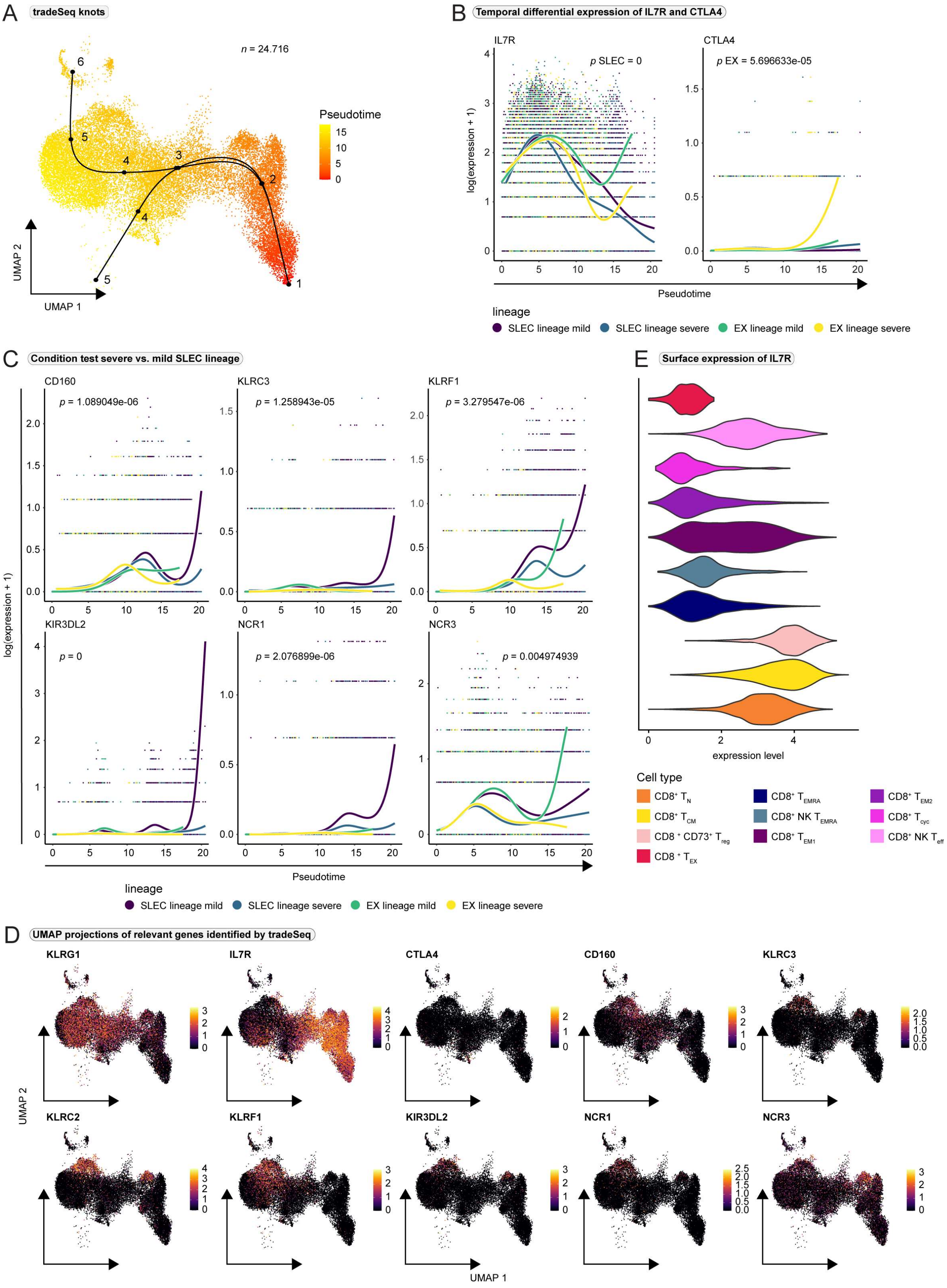

Figure S4. Relevant genes in CD8⁺ T cell differentiation during SARS-CoV-2 infection identified by tradeSeq. (A) tradeSeq knots that subdivide pseudotime into partitions, projected onto the integrated UMAP. **(B)** Smoothed expression of *IL7R* and *CTLA4* across pseudotime with the y-axis on natural logarithmic scale. *p*-values report the result of differential expression

analysis between progenitor and differentiated cell states across pseudotime (start vs. end testing) for the indicated lineage. **(C)** Smoothed expression of selected NK cell-related genes, which are expressed at significantly higher amounts during SLEC differentiation in mild compared to severe COVID-19 (condition testing). **(D)** UMAP projections of relevant genes from *start vs. end testing* and *condition testing*. **(E)** Surface expression of IL7R (CITE-seq) in $CD8^+$ T cell subtypes. SLEC, short-lived effector cells; EX, exhaustion lineage.

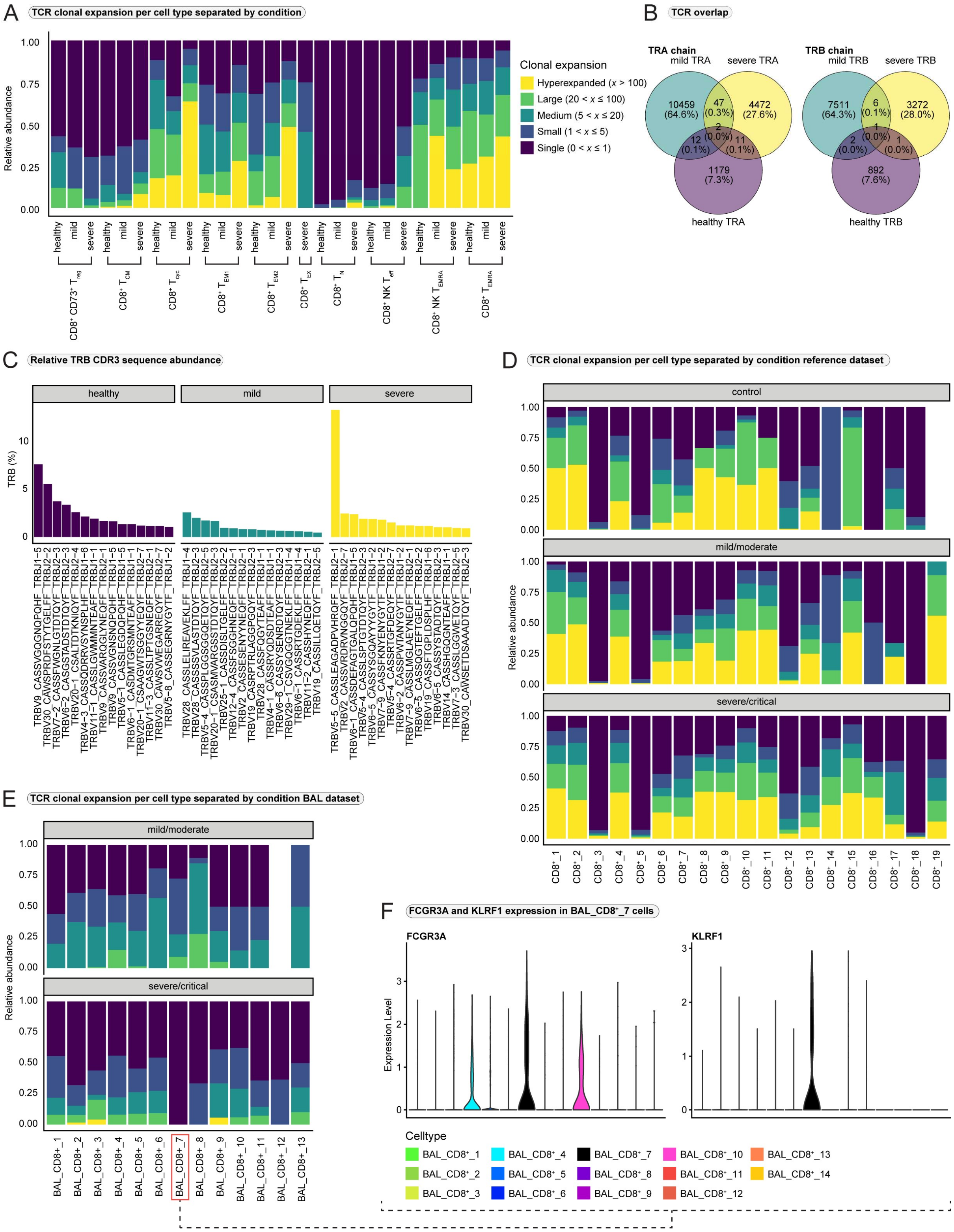

Figure S5. Clonal expansion and TCR diversity in COVID-19. (A) Distribution of clonal expansion per cell type within each condition, displayed as relative abundance of clonotype expansion groupings. **(B)** Overlap in TCR sequences between the conditions for TCR alpha chain (left) and TCR beta chain (right). For comparing TCR sequences the ‘V gene - CDR3 sequence - J gene’ structures of the TCR-sequences were compared between the conditions. **(C)** Relative proportion of CDR3 sequences of the 15 most abundant clones to the total number

of CDR3 sequences per condition for the TRB chain. **(D)** Distribution of clonal expansion per cell type within each condition of the PBMC-derived $DC8^+$ reference dataset, displayed as relative abundance of clonotype expansion groupings. **(E)** Distribution of clonal expansion per cell type within each condition in the BAL-derived $CD8^+$ reference dataset, displayed as relative abundance of clonotype expansion groupings. **(F)** *FCGR3A* and *KLRF1* expression in the BAL-derived $CD8^+$ reference dataset. TCR, T cell receptor; TRA, T cell receptor alpha chain; TRB, T cell receptor beta chain; CDR3, Complementarity determining region 3; BAL, bronchoalveolar lavage.

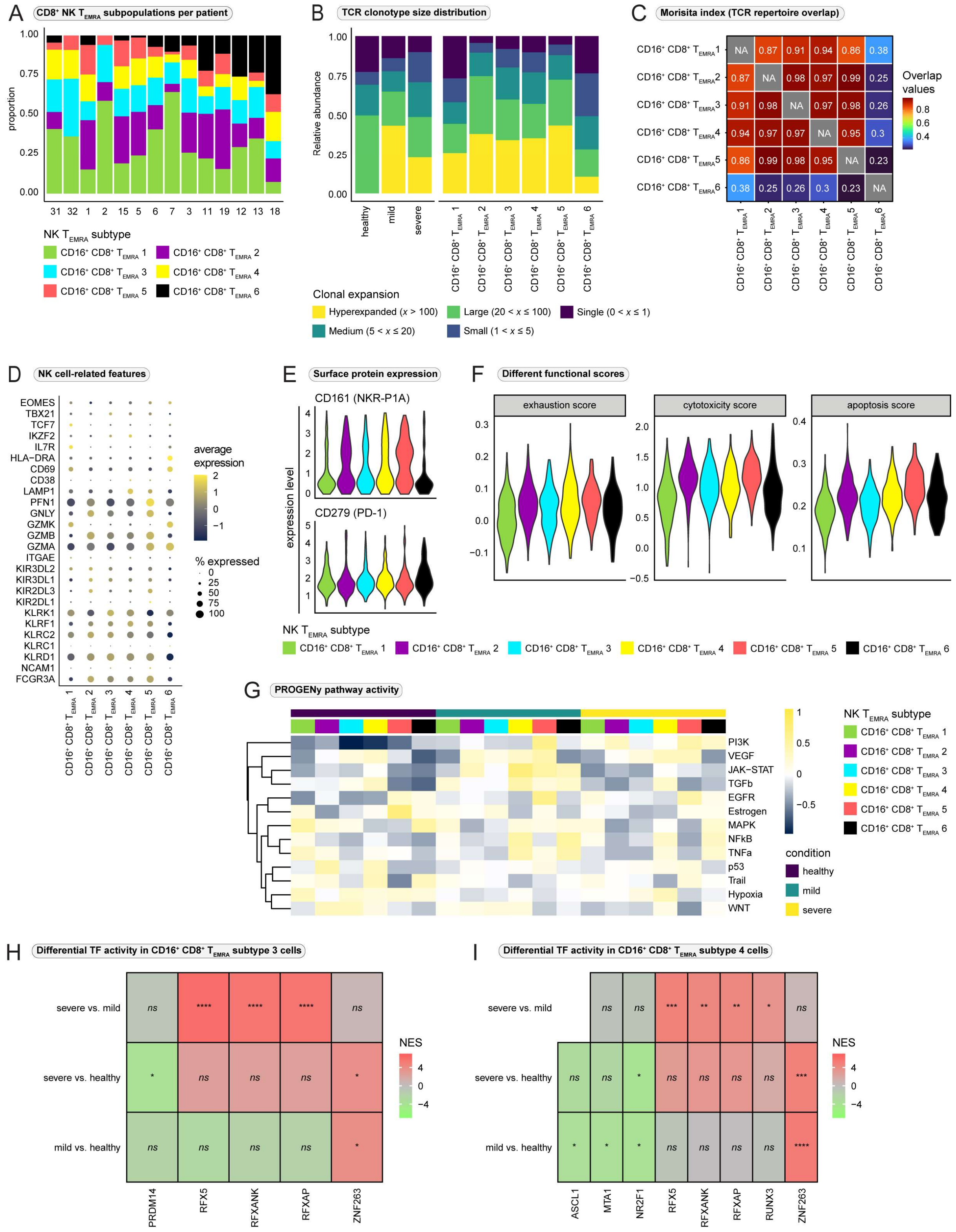

Figure S6. Heterogeneity among CD8$^+$ NK-like terminally differentiated effector memory T cells re-expressing CD45RA. (A) Distribution of CD16$^+$ CD8$^+$ T$_{EMRA}$ subsets per patient. **(B)** Distribution of clonal expansion within the conditions (left) and within the CD16$^+$ CD8$^+$ T$_{EMRA}$ populations (right), displayed as relative abundance of clonotype expansion groupings. **(C)** Overlap in TCR repertoire between the CD16$^+$ CD8$^+$ T$_{EMRA}$ populations estimated with Morisita index. **(D)** Average expression of selected genes related to NK cell phenotype and

function in the six $CD16^+$ $CD8^+$ T_{EMRA} subsets. **(E)** Surface expression (CITE-seq) of CD161 and CD279 per $CD16^+$ $CD8^+$ T_{EMRA} subtype. **(F)** Exhaustion, cytotoxicity and apoptosis scores for the six $CD16^+$ $CD8^+$ T_{EMRA} subsets. **(G)** Pathway activity for all computed PROGENy pathways in $CD16^+$ $CD8^+$ T_{EMRA} cells. **(H)** Differential transcription factor activity (DoRothEA) estimated with *msviper* in $CD16^+$ $CD8^+$ T_{EMRA}-3 cells and **(I)** in $CD16^+$ $CD8^+$ T_{EMRA}-4 cells between the severe and the mild condition. Positive NES values indicate increased activity in severe SARS-CoV-2 infection. TCR, T cell receptor; TF, transcription factor; NES, normalized enrichment score.

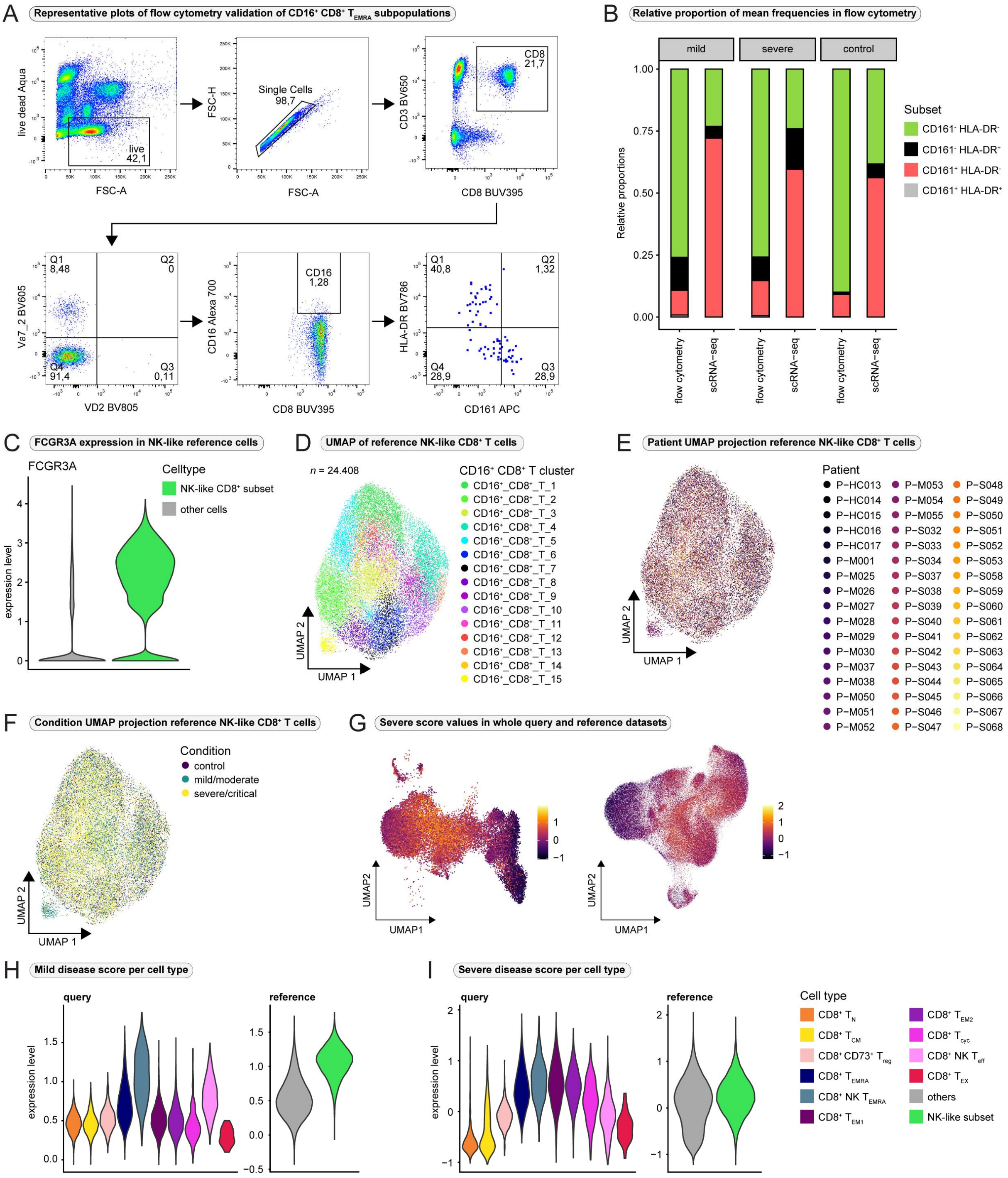

Figure S7. Validation of CD16⁺ CD8⁺ TEMRA cells and mild and severe disease scores. (A) Representative gating strategy of the flow cytometry analysis for the validation of the existence of CD16⁺ CD8⁺ TEMRA subsets. After exclusion of dead cells and doublets, we first gated on CD8⁺ T cells. By gating on TCR Vδ2⁻ and TCR Vα7.2⁻ cells we excluded major populations of γδ T cells and MAIT cells. Finally, we gated on CD16⁺ cells and quantified the frequency of cells expressing different combinations of HLA-DR and CD161. **(B)** Relative proportions of the mean frequencies of CD16⁺ CD8⁺ TEMRA subsets per condition as observed by flow cytometry vs. scRNA-seq. **(C)** *FCGR3A* expression plotted for all cells in the reference CD8⁺ T cell dataset that mapped together with our CD8⁺ NK-like TEMRA cells in the integrated dataset

(NK-like CD8$^+$ subset) and for all other cells in the reference dataset (other cells). **(D)** UMAP embedding of the subclustered NK-like CD8$^+$ T cell subset from the reference dataset (n = 24,408). **(E)** Per patient-origin and **(F)** per condition-origin projected onto the UMAP of the NK-like CD8$^+$ T cell subset from the reference CD8$^+$ T cell dataset. **(G)** Severe disease score values projected onto the UMAP embeddings of our CD8$^+$ T cell dataset (left) and the reference CD8$^+$ T cell dataset (right). **(H)** Mild and **(I)** severe disease score values per CD8$^+$ T cell type in our query dataset (left panels) and the NK-like CD8$^+$ T cell subset compared with all other CD8$^+$ T cells in the reference dataset (right panels).

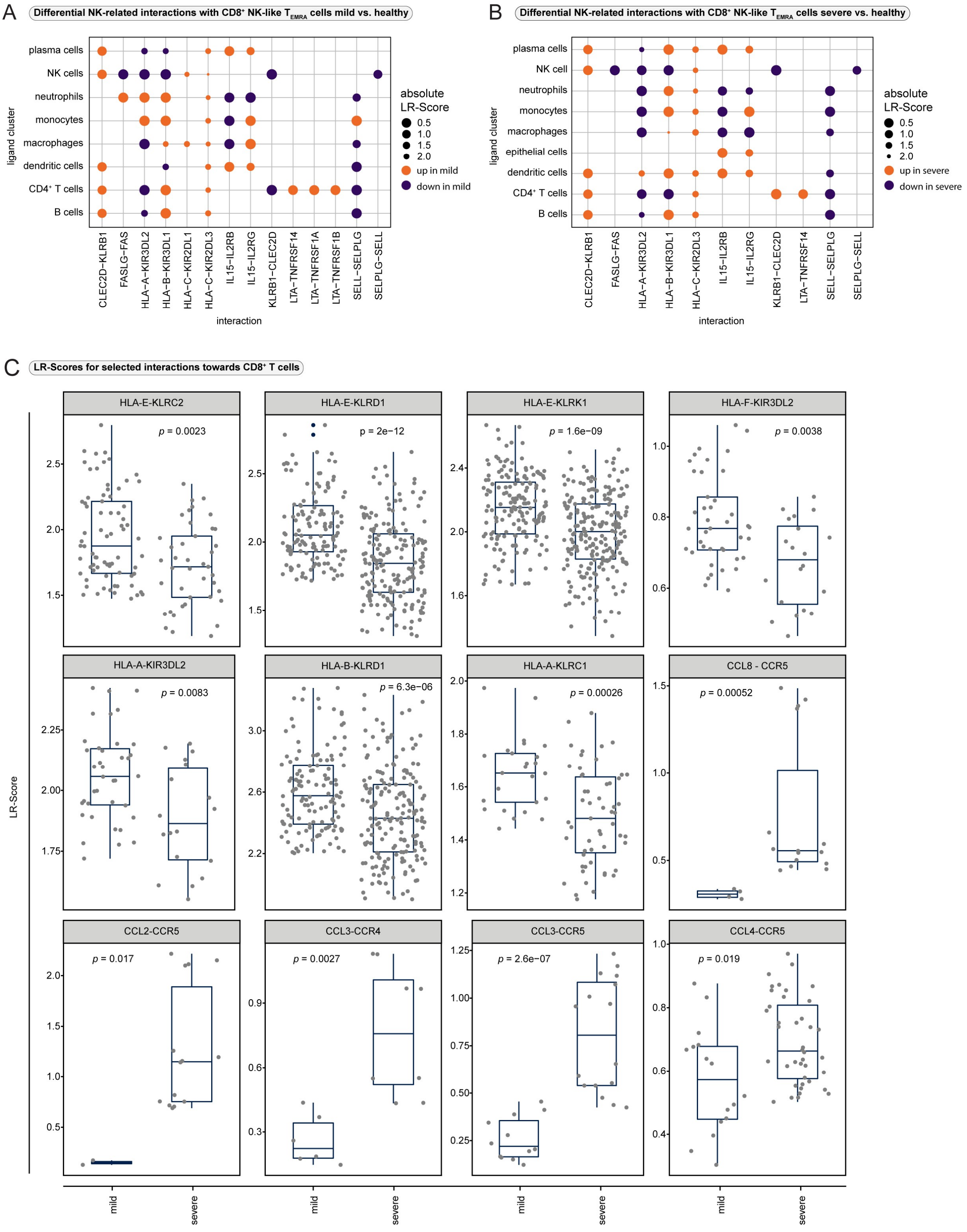

Figure S8. Additional cell-cell interactions. (A) Differential ligand-receptor interactions between mild COVID-19 and healthy controls and **(B)** severe COVID-19 and healthy controls. To assess interactions between CD8$^+$ T cells and non-T cells our dataset was integrated with the whole reference dataset and interactions were predicted using *LIANA*. Differential interactions were then calculated using CrossTalkeR for selected interactions, relevant in NK cell development and function. A group of ligand clusters was selected and NK-like CD8$^+$

T_{EMRA} cells were regarded as receptor cluster. The size of the dots indicates the absolute value of the differential LR-Score. The color indicates the direction of the change in ligand-receptor interactions; orange indicates increased interactions in the disease condition, purple indicates decreased interactions in the disease condition. **(C)** Boxplots displaying differences in LR-Scores for selected NK cell receptor and CC chemokine interactions between severe and mild COVID-19. All $CD8^+$ T cell populations were regarded as receptor population and all other populations (except megakaryocytes) were regarded as ligand population for this purpose. LR-Score, ligand-receptor score.

1.2 Supplementary Tables

Table S1. CITE-Seq antibody panel

Antibody	Source	Catalog number
TotalSeqTM-C0092 Mouse IgG2b, κ isotype Ctrl (Clone: MPC-11)	BioLegend	400381
TotalSeqTM-C0091 Mouse IgG2a, κ isotype Ctrl (Clone: MOPC-173)	BioLegend	400293
TotalSeqTM-C0090 Mouse IgG1, κ isotype Ctrl (Clone: MOPC-21)	BioLegend	400187
TotalSeqTM-C0063 anti-human CD45RA, (Clone: HI-100)	BioLegend	304163
TotalSeqTM-C0088 anti-human CD279 (PD-1), (Clone: EH12.2H7)	BioLegend	329963
TotalSeqTM-C0087 anti-human CD45RO, (Clone: UCHL1)	BioLegend	304259
TotalSeqTM-C0159 anti-human HLA-DR, (Clone: L243)	BioLegend	307663
TotalSeqTM-C0149 anti-human CD161, (Clone: HP-3G10)	BioLegend	339947
TotalSeqTM-C0081 anti-human CD14, (Clone: M5E2)	BioLegend	301859
TotalSeqTM-C0148 anti-human CD197 (CCR7), (Clone: G043H7)	BioLegend	353251
TotalSeqTM-C0390 anti-human CD127 (IL-7Rα), (Clone: A019D5)	BioLegend	351356
TotalSeqTM-C0050 anti-human CD19, (Clone: HIB19)	BioLegend	302265
TotalSeqTM-C0080 anti-human CD8a, (Clone: RPA-T8)	BioLegend	301071
TotalSeqTM-C0072 anti-human CD4, (Clone: RPA-T4)	BioLegend	300567
TotalSeqTM-C0034 anti-human CD3, (Clone: UCHT1)	BioLegend	300479

Table S2. Clinical data

Patient	Sex	Age	ICU	Ventilation	Days since first positive PCR	Status of infection at time of sampling	SARS-CoV-2 IgG antibody test result	Group
P01	female	78	no	no	5	positive	negative	mild
P02	female	77	no	no	7	positive	negative	mild
P03	male	64	yes	yes	12	positive	positive	severe
P05	female	27	no	no	50	negative	negative	mild
P06	male	28	no	no	58	negative	negative	mild
P07	female	25	no	no	57	negative	indeterminate	mild
P11	male	56	yes	yes	57	positive	positive	severe
P12	female	67	yes	yes	55	negative	indeterminate	severe
P13	female	62	yes	yes	49	negative	positive	severe
P15	female	75	no	no	7	positive	indeterminate	mild
P18	male	62	yes	yes	96	negative	positive	severe
P19	male	82	yes	yes	11	positive	indeterminate	severe
P29	female	52	-	-	-	-	negative	healthy
P31	male	38	-	-	-	-	negative	healthy
P32	male	79	-	-	-	-	negative	healthy

ICU = intensive care unit

Tables S3-S9 are available as xlsx-files

Table S10. Samples from the public dataset that were chosen for integration with our dataset for cell-cell interaction analysis

sampleID	PatientID	Condition
S-M074-1	P-M074	mild/moderate
S-S086-1	P-S086	severe/critical
S-M076-1	P-M076	mild/moderate
S-S085-1	P-S085	severe/critical
S-S087-1	P-S087	severe/critical
S-S088-1	P-S088	severe/critical
S-S089-1	P-S089	severe/critical
S-S090-1	P-S090	severe/critical
S-S090-2	P-S090	severe/critical
S-M076-2	P-M076	mild/moderate
S-M074-2	P-M074	mild/moderate
S-S088-2	P-S088	severe/critical
S-S085-2	P-S085	severe/critical
S-S089-2	P-S089	severe/critical
S-S087-2	P-S087	severe/critical
S-S086-2	P-S086	severe/critical
S-HC003	P-HC003	control
S-HC005	P-HC005	control
S-HC010	P-HC010	control
S-HC011	P-HC011	control
S-HC013	P-HC013	control
S-HC014	P-HC014	control

Table S11 is available as xlsx-files
